# Comparison of inhomogeneous Schottky barrier modeling approaches in SiC polytypes

**DOI:** 10.1371/journal.pone.0355480

**Published:** 2026-08-21

**Authors:** Fayssal Mekaret, Abdelaziz Rabehi, Baya Zebentout, Shahrazade Tizi, Abdelmalek Douara, Mawloud Guermoui, Imad Eddine Tibermacine, Amal H. Alharbi, El-Sayed M. El-kenawy, Mustapha Habib, Zineb Benamara

**Affiliations:** 1 Applied Microelectronics Laboratory (AMEL), Electronics Department, Faculty of Technology, Djillali Liabes University of Sidi Bel Abbes, Sidi Bel Abbes, Algeria; 2 Telecommunication and Smart Systems Laboratory, Faculty of Sciences and Technology, Ziane Achour University, Djelfa, Algeria; 3 Department of Computer, Control and Management Engineering, Sapienza University of Rome, Italy; 4 Department of Computer Sciences, College of Computer and Information Sciences, Princess Nourah Bint Abdulrahman University, Riyadh, Saudi Arabia; 5 Delta Higher Institute of Engineering and Technology, Department for Communications and Electronics, Mansoura, Egypt; 6 Jadara Research Center, Jadara University, Irbid, Jordan; 7 KTH Royal Institute of Technology, Civil and Architectural Engineering, Teknikringen 78, Stockholm, Sweden; Universidad Autónoma de Querétaro: Universidad Autonoma de Queretaro, MEXICO

## Abstract

This work presents a comprehensive comparative analysis of Schottky barrier (SB) inhomogeneities in three SiC polytypes (3C, 4H, and 6H) using three established theoretical approaches: the Cowley and Sze interface state model, the Werner Gaussian distribution model, and the Tung localized patch model. Through analytical simulations performed in MATLAB, we examined how each model’s intrinsic parameters, including the interface state density (N_ss_), the standard deviation (σ), and the inhomogeneity parameter (γ), influence the effective Schottky barrier height (SBH) and the corresponding I–V characteristics predicted by each model. Our results demonstrate that each model reveals a distinct physical mechanism governing charge transport at the metal/semiconductor interface. The Cowley and Sze model emphasizes Fermi-level pinning due to interface states, Werner’s model accounts for statistical fluctuations of the barrier height, and Tung’s model describes localized current flow through low-barrier patches. Even though the numerical values obtained for each case are different, Although the three SiC polytypes exhibit different quantitative responses, they all show thermally activated transport behavior. These findings provide valuable guidelines for optimizing Schottky contact performance in high- temperature SiC power devices. The proposed comparative framework serves as a predictive tool for designing, engineering, and improving metal/SiC interfaces in advanced power device technologies.

## 1. Introduction

Silicon carbide (SiC) has emerged as one of the most promising wide-bandgap semiconductors for high-power, high-frequency, and high-temperature electronic applications due to its large bandgap, high breakdown electric field, excellent thermal conductivity, and outstanding chemical stability [1].

These properties make SiC particularly suitable for next-generation electronic devices intended to operate under extreme conditions, where conventional silicon based technologies reach their physical limits [2]. Among the various types of metal-semiconductor contacts, the Schottky contact plays a decisive role in determining device performance, especially in fast rectifiers and high-voltage components [3].

In the ideal theory of Schottky contacts, the barrier height at the metal-semiconductor interface is assumed to be spatially uniform and determined by the difference between the metal work function and the semiconductor electron affinity, as described by the Schottky–Mott model [4]. However, numerous experimental studies on SiC-based Schottky diodes have revealed significant deviations from this ideal behavior. In practice, the Schottky barrier height (SBH) often exhibits spatial inhomogeneity arising from interface states, structural defects, surface roughness, interfacial layers, as well as local variations in doping and composition. These inhomogeneities strongly affect carrier transport mechanisms, leading to temperature-dependent effective barrier heights, non-ideal current–voltage characteristics, and deviations from classical thermionic emission theory [5].

To explain these behaviors, several theoretical models have been developed to describe Schottky barrier inhomogeneity.

**Fig 1:**
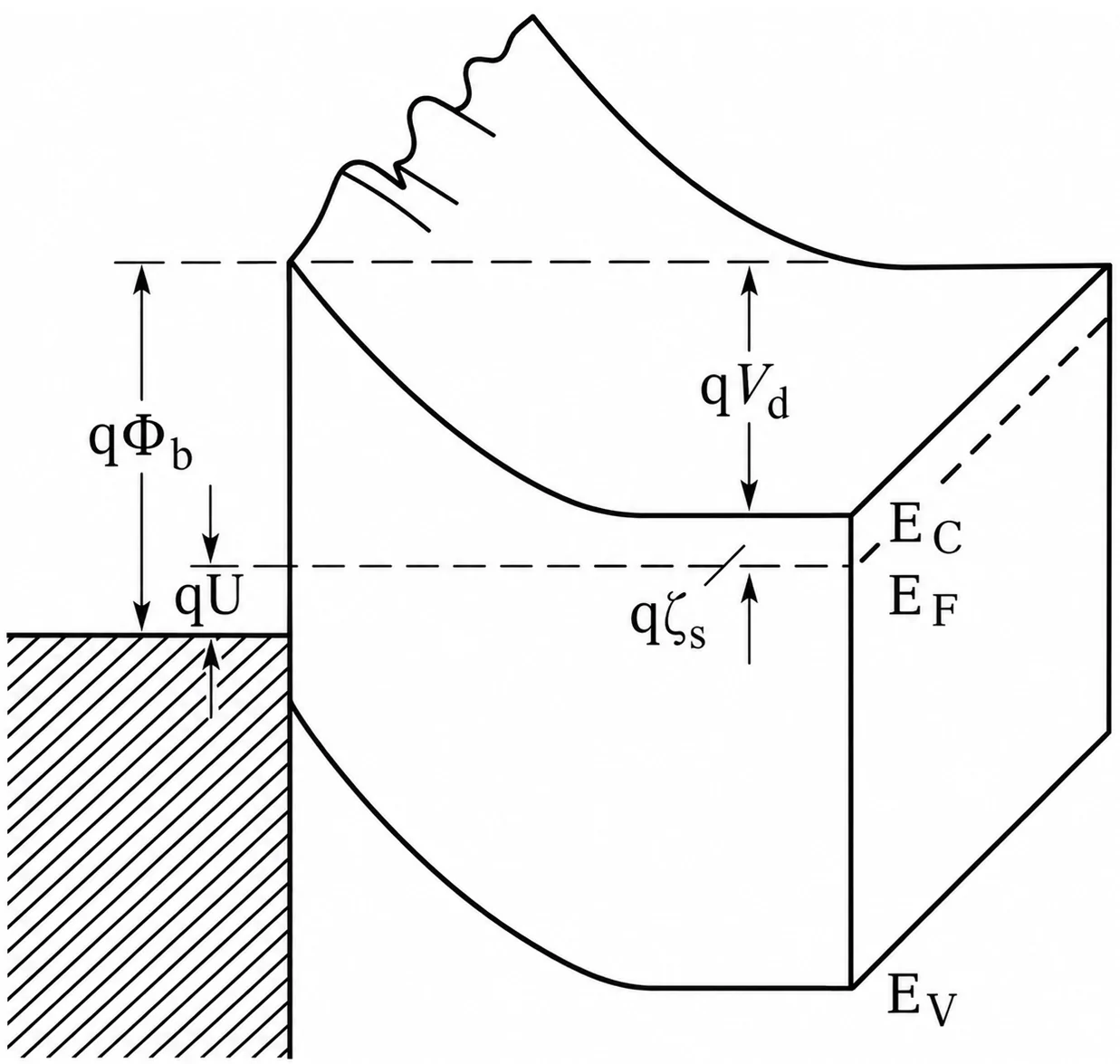
Two-dimensional band diagram of an inhomogeneous Schottky barrier [7].

The interface state model proposed by Cowley and Szekn attributes barrier modification to Fermi-level pinning induced by a high density of interface states at the metal-semiconductor junction [6]. Subsequently, Werner and co-workers introduced a statistical model based on a Gaussian distribution of barrier heights, enabling the interpretation of experimentally observed temperature-dependent electrical characteristics [7]. The schematic representation of this inhomogeneous barrier model is shown in Fig 1.

Complementarily, the localized patch model proposed by Tung assumes that current transport is dominated by local low-barrier regions embedded within a higher barrier background, providing a physical interpretation of spatially non-uniform interfaces [8].

**Fig 2:**
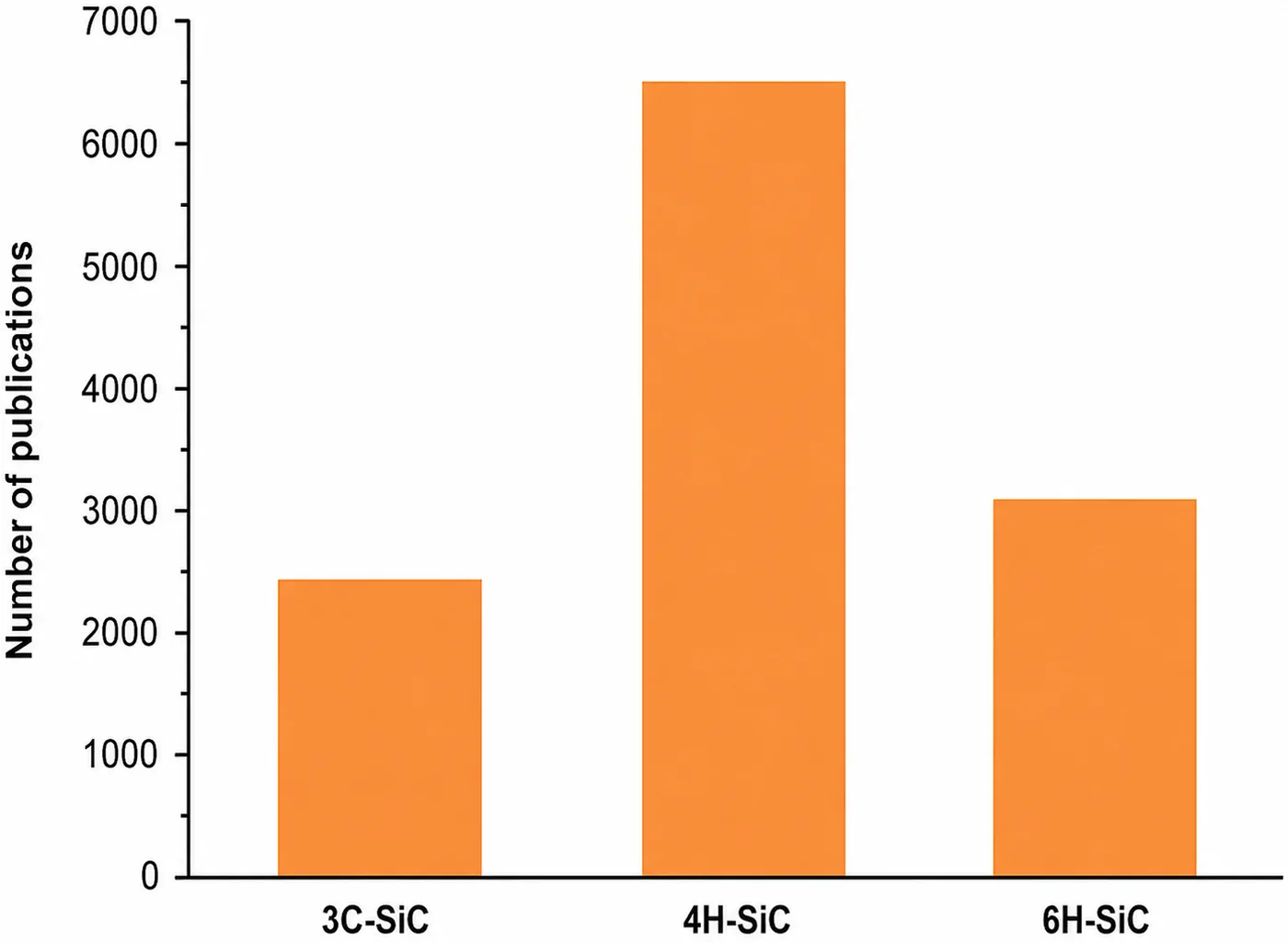
Total number of SiC publications. Data are extracted from the Web of Sci-ence Core Collection with keywords “3C-SiC”, “4H-SiC” and “6H-SiC” [9].

Most published studies on SiC-based Schottky diodes focus either on a specific inhomogeneity model or on a single SiC polytype, particularly 4H-SiC, which currently dominates commercial power electronics applications [9]. The statistics of published works concerning SiC are illustrated in Fig 2.

In this context, the present work provides a comprehensive comparative study of Schottky barrier inhomogeneity in three SiC polytypes (3C-SiC, 4H-SiC, and 6H-SiC) based on three widely recognized theoretical frameworks: the Cowley–Sze interface-state model, the Werner Gaussian distribution model, and Tung’s localized patch model.

Using analytical simulations performed in MATLAB, we investigate the influence of key inhomogeneity parameters on the effective barrier height and current–voltage characteristics under identical physical conditions.

Although the Cowley–Sze, Werner, and Tung models are widely used to analyze Schottky barrier inhomogeneities, comparative studies examining these approaches under identical simulation conditions and across different SiC polytypes remain limited.

The novelty of this work lies in the development of a unified analytical simulation platform that confronts the interface-state model, the Gaussian inhomogeneity model, and the microscopic patch theory under identical physical boundaries.

By correlating the interfacial parameters (σ, γ, and Nss) with the intrinsic properties of 3C-SiC, 4H-SiC, and 6H-SiC, this study provides a comparative framework for evaluating the influence of barrier inhomogeneities on charge transport behavior. The obtained results help clarify the applicability of each model under different interface conditions and SiC polytypes.

The rest of the paper is organized as follows. Section 2 presents the theoretical models and simulation parameters used in this study. Section 3 discusses the comparative results, sensitivity analyses, and experimental validation for the different SiC polytypes. Finally, Section 4 summarizes the main conclusions and engineering insights.

## 2. Analytical models for Schottky Barrier Inhomogeneity

### 2.1. Homogeneous case (ideal case)

In an ideal metal-semiconductor junction, the SBH ${(\Phi }_{Bideal})$ can be accurately estimated using the Schottky-Mott rule, originating in the 1930s, this rule applies electrostatic principles to predict energy-level alignment in various problems [10].

According to the Schottky-Mott model, $\Phi_{Bideal\;}$ is expressed from the difference between the metal work function and the semiconductor affinity


$$\begin{array}{c} \Phi_{Bideal\;}=\Phi_{M\;}-\chi \end{array}$$ (1)

### 2.2. Inhomogeneous case

In an ideal Schottky contact, the barrier height at the metal–semiconductor interface is considered uniform across the entire junction area. However, in real devices, this assumption often fails due to various imperfections at the interface, such as surface roughness, defects, non-uniform doping, and interfacial layers [11].

These factors lead to spatial fluctuations in the local barrier height, resulting in what is referred to be an inhomogeneous SB. Instead of a single, well-defined barrier, the contact exhibits a distribution of barrier heights.

This inhomogeneity significantly influences the electrical behavior of the device, particularly at low temperatures or under small forward biases, where carriers preferentially traverse regions of lower barrier height [12].

Several models have been developed to describe this phenomenon, including the interface-state model (Cowley and Sze), the Gaussian distribution model (Werner), and the localized patch model (Tung), each offering different insights into the nature and impact of the inhomogeneities.

#### 2.2.1. The Cowley and Sze model.

The Cowley and Sze theory describes the formation of the SB in the presence of a high density of interface states at the metal–semiconductor junction [6].

These states pin the Fermi level at the interface, making the SBH largely independent of the metal work function [13].

This model contrasts with the ideal case (Schottky–Mott model), which assumes a clean interface without interface states.


$$\begin{array}{c} \Phi_{𝐁\;𝐂𝐨𝐰𝐥𝐞𝐲}=\;C_{2}\left(\;\Phi_{𝐌-}\;\chi_{𝐬𝐜}\right)+\left(1-C_{2}\right)\left({\;𝐄}_{𝐠}-\;{\;\Phi }_{0}\;\right) \end{array}$$ (2)

$\Phi_{0}$ represents the neutral level of interface states, and is given by:


$$\begin{array}{c} \Phi_{0}=\frac{𝐄𝐠}{𝐪}-\;\frac{{𝐂}_{2\;}\chi +\;{𝐂}_{3\;}}{1-{𝐂}_{2\;}} \end{array}$$ (3)

$$\begin{array}{c} {𝐍}_{𝐬𝐬}=\frac{\left(1-{𝐂}_{2}\;\right)\epsilon_{𝐬}}{{𝐂}_{2}{𝐝}_{ox}.{𝐪}^{2}} \end{array}$$ (4)

$N_{ss}$ and $d_{ox}$ denote the density of interface states and the thickness of the interfacial oxide layer, respectively, while${\;\epsilon }_{𝐨𝐱}$ represents the permittivity of the semiconductor.

The values of ${\text{C}}_{\text{2}}$ and ${\text{C}}_{\text{3}}\;$ are extracted from the $\Phi_{\text{B}}$ curve as functions of $\;\Phi_{\text{M}}$ for each polytype. Here, ${\text{C}}_{\text{2}}$ represents the slope, and ${\text{C}}_{\text{3}}$ denotes the intercept. The values of ${\text{C}}_{\text{2}}$ and ${\text{C}}_{\text{3}}$ are discussed in detail in our previous work [14].

#### 2.2.2. The Gaussian model (Werner model).

After Cowley and Sze model, we examine the Gaussian distribution approach is examined to describe barrier inhomogeneities in Schottky junctions.

This model assumes that the barrier height follows a Gaussian distribution, with variations around an average value [15].

The standard deviation σof this distribution characterizes the degree of inhomogeneity in the barrier [16], and this factor plays a crucial role in determining the electrical properties of the junction.

According to the Werner model, the total current density J flowing through the Schottky diode is given by:


$$\begin{array}{c} \;J=\int j\left(V,\Phi_{B\;}\right)\rho \left(\Phi_{B\;}\right)d\Phi_{B\;} \end{array}$$ (5)

$j\left({V,\Phi }_{B\;}\right)$is the density of the elementary thermionic current emitted in the Schottky diode with barrier $\Phi_{B}$, and $\rho \left(\Phi_{B\;}\right)$ is the Gaussian distribution


$$\begin{array}{c} j\left({V,\Phi }_{B\;}\right)=J_{s}\left[exp\left(\frac{qV_{a}}{k_{B}T}\right)-1\right] \end{array}$$ (6)

Where J_s_ is the saturation current:


$$\begin{array}{c} J_{s}=AA^{*}T^{2}exp\left(\frac{-q\Phi_{Beffe}}{k_{B}T}\right) \end{array}$$ (7)

$$\begin{array}{c} \rho \left(\Phi_{B},\sigma \right)=\frac{1}{\sqrt{2\pi \sigma^{2}}}\;exp\;\left[-\frac{{(\Phi_{B\;}-\Phi_{B0\;})}^{2}}{{2\sigma }^{2}}\right] \end{array}$$ (8)

$$\begin{array}{c} \Phi_{effe}={\overset{―}{\Phi }}_{B0\;}-\frac{q\sigma^{2}}{2k_{B}T} \end{array}$$ (9)

The lower integration limit $\Phi_{Bmin}$ is equal ${\overset{―}{\Phi }}_{B0\;}-7\sigma$ and the maximum integration limit is equal ${\overset{―}{\Phi }}_{B0\;}$ [17] which is the average value of the barrier and is equivalent to the ideal barrier in the homogeneous case (the difference between the metal’s work function and the semiconductor’s electron affinity). $\Phi_{effe}$ denotes the corresponding effective barrier**.** The maximum Schottky barrier height ${\overset{―}{\Phi }}_{B0\;}$ as well as the variance σ are suggested in the literature to be in principle dependent on the applied bias V [18].

#### 2.2.3. Tung model.

The Tung model describes Schottky barrier inhomogeneity by assuming the presence of localized low-barrier patches embedded within a higher uniform barrier background [19].

These patches arise from microscopic interface defects or non-uniformities and dominate current transport at low temperatures. Unlike statistical models, Tung’s approach provides a physical interpretation of inhomogeneity based on patch geometry and distribution, introducing an inhomogeneity parameter γ that quantifies the effect of these regions on the overall device behavior [19], γ is defined as:


$$\begin{array}{c} \gamma =\text{3}{\left(\frac{\Delta R_{0}^{2}}{4}\right)}^{1/3} \end{array}$$ (10)

The equation (10) relates γ to Δ the barrier lowering (the difference between the high barrier and the local low barrier), and R_0_, the radius of the patch.

A larger Δ or R_0_ increases γ, indicating stronger inhomogeneity and a greater contribution of the patch to the total current.

The formulation of the current in a low-barrier region is as follows:


$$\begin{array}{c} I_{patch}=A^{*}\;A_{patch}𝐞𝐱𝐩\left(-\beta \Phi_{effe\;}\right)exp\left(\beta \;V_{a}-1\right) \end{array}$$ (11)

$$\begin{array}{c} A_{patch}=4\pi \gamma \eta^{2/3}/9\beta V_{bb}^{2/3} \end{array}$$ (12)

$$\begin{array}{c} \Phi_{Beffe}=\Phi_{B0\;}-\frac{\gamma V_{bb}^{1/3}}{\eta^{1/3}} \end{array}$$ (13)

Where $\;A_{patch}$ is the effective area of current conduction for the patches and $\Phi_{effe\;}$is the effective barrier height, $V_{bb}$ is the band bending under forward bias $V_{a}$. $\Phi_{B0\;}$is the ideal barrier (homogeneous case) [20].


$$\begin{array}{c} V_{bb}=\Phi_{M}-\left(\chi +\frac{kT}{q}\times log\frac{N_{c}}{N_{d}}\;\right) \end{array}$$ (14)

$$\begin{array}{c} \eta =\frac{2\varepsilon_{s}}{qN_{d}} \end{array}$$ (15)

$N_{d}$ is the doping concentration.

Considering a narrow distribution of γ, and assuming a patch density of $c_{1}$, the total current flowing through the individual patches is given by:


$$\begin{array}{c} \sum I_{patch}=c_{1}AI_{patch\;} \end{array}$$ (16)

Where A is the total diode area.

The current through the high barrier, bulk region of the diode area is given by the thermionic emission equation:


$$\begin{array}{c} I_{bulk}=A\left({1-c}_{1}A_{patch}\right)A^{*}T^{2}𝐞𝐱𝐩\left(-\beta \Phi_{B0\;}\right)exp\left(\beta V_{a}-1\right) \end{array}$$ (17)

The total current flowing through the diode is determined as the sum of the low-barrier patch current ($I_{patch}$) and the current passing through the uniform bulk region ($I_{bulk}$) with a barrier height of $\Phi_{B0}$ [20].


$$\begin{array}{c} I_{tot}=AA^{*}T^{2}𝐞𝐱𝐩\left(-\beta \Phi_{B0\;}\right)exp\left(\beta V_{a}-1\right)\;[1+\frac{4c_{1}\pi \eta^{2/3}\;\gamma }{9\beta V_{bb}^{2/3}}𝐞𝐱𝐩(\frac{\beta \gamma V_{bb}^{\frac{1}{3}}}{\eta^{\frac{1}{3}}})] \end{array}$$ (18)

## 3. Simulation results and discussion

Before starting our analytical simulation, in the study of inhomogeneous Schottky barriers in the three SiC polytypes, it is crucial to select the metal that ensures good rectifying behavior for each polytype.

To select the most suitable junction metal for each SiC polytype, we relied on previously published experimental studies dedicated to the investigation of the Schottky barrier for each specific polytype.

It is worth noting that 4H-SiC is the most widely used polytype in reported SiC-based Schottky diodes. This material has demonstrated remarkable electrical stability and high compatibility with a broad range of contact metals, including titanium, nickel, tungsten, and molybdenum. The choice of metal for each polytype is based on the following studies:

F. Roccaforte et all [21], J. Eriksson [22] et all and G. Constantinidis et all [23] demonstrated through detailed experimental studies that Platinum, when used as a junction metal with 3C-SiC, offers excellent Schottky rectification performance.

J.M. Bluet et all [24] and M. E. Aydın [25] have published experimental studies demonstrating the very good compatibility between Nickel and 4H-SiC. When these two are brought into contact, the resulting Schottky diode exhibits excellent rectification performance.

M. O. Aboelfotoh [26] and A.Bekaddour [27]et all investigated the Titan/6H-SiC Schottky diode, and the published results demonstrate the efficiency and high performance of this device. It should be noted that the doping level was fixed at 10¹⁵ cm ⁻ ³ throughout the entire simulation.

The simulations were carried out in MATLAB using the analytical formulations of the Cowley–Sze, Werner, and Tung models over the temperature range of 100–500 K. For each temperature, the effective Schottky barrier height was calculated using the corresponding model equations and the material parameters listed in Table 1. The forward current density was then determined over the voltage range from 0 to 3V by solving the thermionic-emission equation including the series resistance term.

**Table 1:** Experimental values of the ideality factor at different temperatures.

Diode	n for 100K	n for 200K	n for 300K	n for 400K	n for 500K	Reference
Pt/3C-SiC	/	1.4	1.22	1.17	1.14	[21], [22]
Ni/SiC	2.04	1.31	1.17	1.11	1.08	[24], [25]
Ti/6H-SiC	1.89	1.24	1.11	1.06	1.03	[26], [27]

The numerical solution was obtained using the Newton–Raphson iterative method. The iteration process was repeated until the absolute difference between two successive current-density values became smaller than 10^−12^ A/cm^2^.

A maximum number of iterations was imposed to ensure numerical stability. All calculations were performed using double-precision arithmetic. The simulations assume one-dimensional transport and uniform material properties for each SiC polytype.

The numerical implementation was validated by verifying the physical consistency of the results and their agreement with trends reported in the literature

### 3.1. Study of the Schottky barrier for each model

#### 3.1.1. Cowley and Sze SB.

In this first part, we investigate the relationship between three critical parameters of the inhomogeneous barrier according to The Cowley and Sze model: temperature, ideality factor(n), and interface states (N_ss_).

The variation of the calculated interface state density across the studied structures reflects the degree of Fermi-level pinning. Physically, a higher value of N_ss_ indicates a greater density of structural defects at the interface, these defects act as an electrostatic screen against the metal work function [6]. Consequently, as N_ss_ increases, the control of the contact metal over the Schottky barrier height diminishes, pinning the Fermi level near the charge neutrality level ($\Phi_{0}$) [10].

This pinning effect varies with the selected SiC polytype due to their distinct surface energies and band structures, shifting the transport dynamics from an ideal case to a surface-state dominated regime [28].


$$\begin{array}{c} n=1+\frac{d_{ox}}{\varepsilon_{i}}\;\left[\frac{\varepsilon_{s}}{w}+qN_{ss}\right] \end{array}$$ (19)

Wis the width of the depletion zone.


$$\begin{array}{c} 𝐖=\sqrt{\frac{2\epsilon_{𝐬}}{𝐪{𝐍}_{𝐝}}\;({𝐯}_{𝐛𝐛}-V_{a}-\frac{𝐤𝐓}{𝐪})} \end{array}$$ (20)

**Fig 3:**
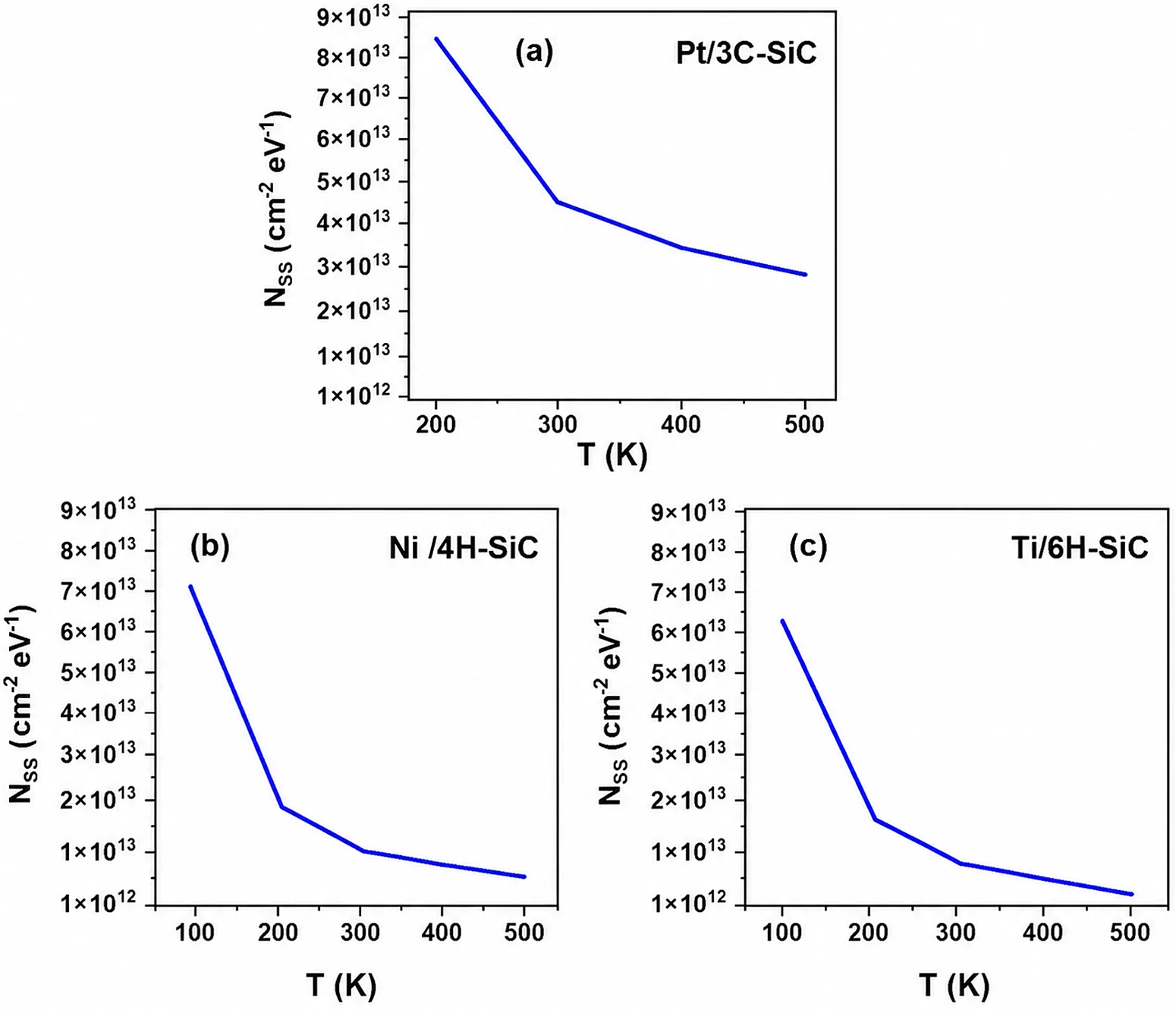
Temperature-Dependent Evolution of Interface States in Schottky Diodes Based on (a) 3C-SiC, (b) 4H-SiC, and (c) 6H-SiC under the Inhomogeneous Barrier Model of Cowley and Sze.

As illustrated in Fig 3, the interface state density N_ss_ exhibits a clear decreasing trend with increasing temperature across all three SiC polytypes.

This physical behavior is in rigorous agreement with the experimental findings of Benamara et al. [29], who observed a similar reduction in N_ss_ for SiC Schottky diodes and attributed it to the thermal emission of trapped charges at the interface.

Furthermore, our results are consistent with the numerical simulations performed under Silvaco by Toumi et al. [30] for Au/n-GaAs Schottky structures, which also demonstrated that interface state effects and barrier inhomogeneities are strongly attenuated as thermal energy rises.

The convergence between our analytical MATLAB simulations and these documented experimental and numerical studies confirms the validity of the proposed framework in capturing the temperature-dependent reorganization of the metal-semiconductor interface

**Fig 4:**
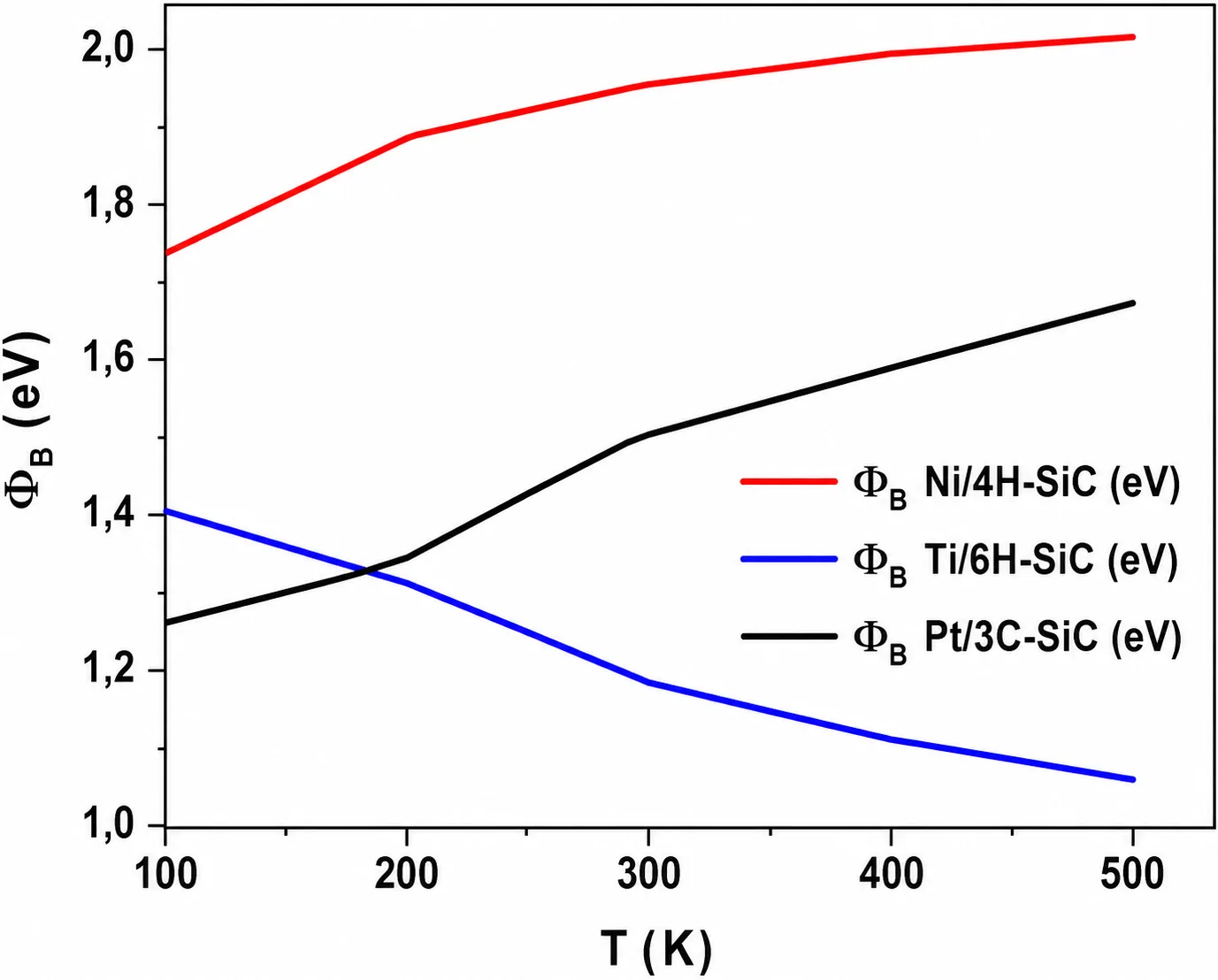
Variation of the Schottky barrier height as a function of temperature for Pt/3C-SiC, Ni/4H-SiC, and Ti/6H-SiC structures based on Cowley and Sze model.

**Fig 5:**
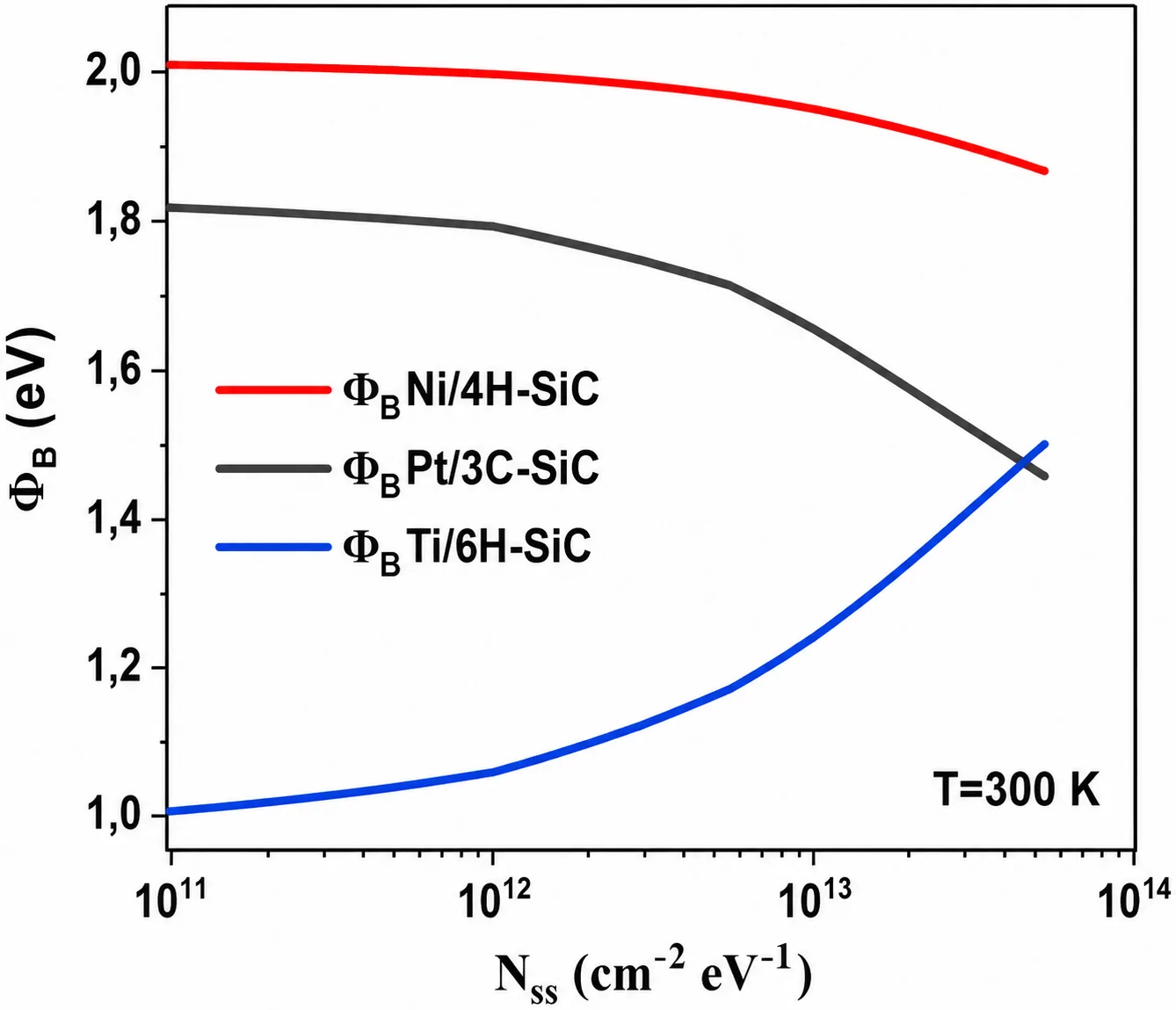
Variation of the Schottky barrier height as a function of interface sates for Pt/3C-SiC, Ni/4H-SiC, and Ti/6H-SiC structures based on Cowley and Sze model.

To better understand the nature of the barrier in the inhomogeneous Cowley and Sze case, it is plotted as a function of temperature and N_ss_ (Figs 4 and 5).

Fig 4 illustrates the evolution of the effective barrier height as a function of temperature for different SiC polytypes.

The N_ss_ values applied in the barrier height calculation are the same as those presented in Fig 3, these values are derived from experimental studies, the references of which are listed in Table 1.

The interfacial layer thickness was fixed at 20 Å (≈ 2 nm) in order to represent realistic metal/SiC interfaces. In practical Schottky contacts, a thin interfacial oxide or disordered transition layer is almost inevitably formed during surface preparation and metal deposition processes [31].

A finite interfacial layer thickness is essential for reproducing non-ideal interface behavior, including interface dipoles, partial Fermi-level pinning, and spatial Schottky barrier inhomogeneity as described by the Cowley–Sze model [6].

For 3C-SiC and 4H-SiC, the effective barrier height increases gradually with temperature, indicating a reduction of the interface-related pinning effects.

As the surface charge density N_ss_ decreases at higher temperatures, the metal/semiconductor coupling becomes stronger, leading the barrier height to approach the intrinsic value defined by the difference between the metal work function and the semiconductor electron affinity.

In contrast, the 6H-SiC polytype shows a clear decrease in the effective barrier height with increasing temperature. In the Cowley and Sze model, the temperature dependence of the barrier height originates from how the two terms in the equation (2) respond to the reduction of the bandgap E_g_(T).

The term $C_{2}\left(\;\Phi_{𝐌-}\;\chi_{𝐬𝐜}\right)$ is largely temperature-independent, while the term $\left(1-C_{2}\right)\left({𝐄}_{𝐠}-\Phi_{0}\right)$ decreases as E_g_ shrinks with increasing temperature.

When $\left(\;\Phi_{𝐌-}\;\chi_{𝐬𝐜}\right)$ is large, the first term dominates and remains stable, making the decrease in the second term too small to affect the overall trend; thus, the barrier height slightly increases with temperature.

Conversely, if $\left(\;\Phi_{𝐌-}\;\chi_{𝐬𝐜}\right)$is small, as in the Ti/6H-SiC contact, the barrier height is primarily governed by $\left({𝐄}_{𝐠}-\Phi_{0}\right)$. Since this term decreases significantly with E_g_(T), the barrier height decreases as temperature rises.

Fig 5 illustrates how the effective SBH evolves as a function of interface state density N_ss_ for three different metal/SiC combinations, based on the Cowley and Sze model. The Fig reveals two distinct trends depending on the specific metal/semiconductor pair.

The selected range of interface state density (N_ss_) in this study spans from 5×10^11^ to 5×10^13^ cm^-2^ eV^-1^. This wide range is physically justified by and closely aligned with the experimental findings reported for 4H-SiC Schottky interfaces.

In particular, the experimental work of Khanna et al [32] on metal/4H-SiC structures demonstrated extracted N_ss_ values ranging between 2×10^12^ to 6.5×10^12^ cm^-2^ eV^-1^ and Benamara et al., who worked on the Ni/6H-SiC diode, reported a reduction in the interface state density N_ss_ from 1.2 × 10^13^ to 6 × 10^12^ cm^−2^ eV^−1^.

By adopting a broader interval 5×10^11^ to 5×10^13^ cm^-2^ eV^-1^, our simulation covers experimental values for both high-quality interfaces and more disordered contacts typically observed in practical SiC devices.

For the Pt/3C-SiC and Ni/4H-SiC contacts, the barrier height decreases progressively as N_ss_ increases. This behavior is attributed to interface states, which disturb the ideal energy alignment at the metal/semiconductor interface.

As the interface-state density increases, the Fermi level becomes more strongly pinned near the charge neutrality level of the semiconductor. When this level lies deeper within the bandgap than the ideal barrier expected from vacuum-level alignment, the overall barrier height is reduced [33].

Conversely, in the Ti/ 6H-SiC structure, the barrier height increases with N_ss_. This indicates that, for this particular combination, the charge neutrality level is located closer to the conduction band edge, and as interface states accumulate, they push the Fermi level upward.

Fundamentally, the interface states serve to amplify the blocking effect, thereby rendering electron injection increasingly difficult as the density of surface states (N_ss_) rises [6].

These contrasting behaviors emphasize the critical role of the semiconductor’s band structure and electron affinity in determining how interface states affect the Schottky barrier. The same mechanism interface-induced band bending can either lower or raise the barrier depending on the energetic alignment between the metal and the semiconductor.

#### 3.1.2. Werner SB.

In this section, we will investigate the effect of temperature and the standard deviation on the Gaussian inhomogeneous effective SB for the three SiC-based polytype diodes.

**Fig 6:**
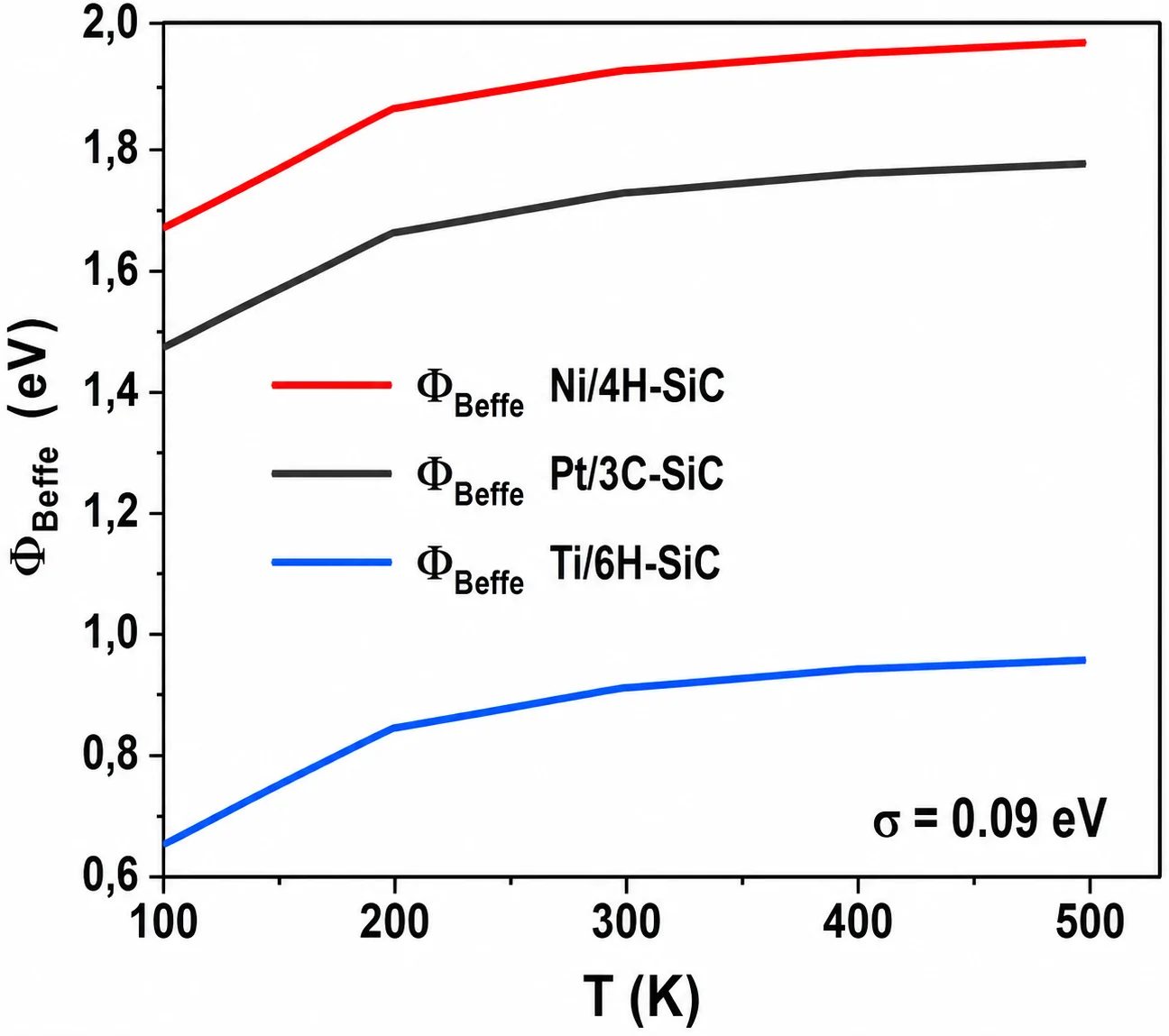
Variation of the Schottky barrier height as a function of temperature for Pt/3C-SiC, Ni/4H-SiC, and Ti/6H-SiC structures based on Werner model.

Fig 6 illustrates the evolution of the effective SBH as a function of temperature for three metal/SiC contacts using Werner’s inhomogeneous barrier model.

To understand the physical origin of the temperature-dependent electrical characteristics, Werner’s model introduces a macroscopic statistical approach where the Schottky barrier height is spatially inhomogeneous and follows a continuous Gaussian distribution [7].

Physically, the current transport is governed by a competitive trade-off between the available thermal energy and the magnitude of potential fluctuations, quantified by the standard deviation (σ) [34].

At high temperatures, charge carriers have enough thermal energy to overcome the mean barrier height. As the temperature decreases, however, carriers preferentially flow through localized regions with lower barrier heights within the Gaussian distribution [35]. Consequently, the experimentally extracted effective barrier height decreases at low temperatures.

This behavior is strongly affected by the intrinsic properties of the SiC polytypes. Wide-bandgap polytypes such as 4H-SiC (Eg ≈ 3.26 eV) are more sensitive to barrier-height fluctuations than narrower-bandgap polytypes such as 3C-SiC (Eg ≈ 2.3 eV). This behavior arises from the enhanced sensitivity of high-barrier contacts to local potential fluctuations [36].

In the numerical of Werner’s model, the standard deviation (σ), which characterizes the degree of barrier height inhomogeneity, was set to **0.09 eV**. This value was carefully selected based on a comparative analysis of established experimental literature on SiC-based Schottky diodes. Specifically, reported extractions of σ for various SiC interfaces **0.09 eV** (Bluet et al. [24]), and **0.092 eV** (Toumi et al [37]) and **0.08 eV** (Ouennoughi et al [38]). By adopting a mean value of 0.09 eV, we ensure that our simulation framework remains within a physically realistic range, accurately reflecting the potential fluctuations typically observed in real-world SiC power devices.

At low temperatures, electrons do not possess sufficient thermal energy to overcome high-barrier regions. As a result, current transport is dominated by low-barrier regions, leading to a lower effective barrier height [39].

With increasing temperature, charge carriers acquire sufficient energy to overcome higher barrier regions. Consequently, current transport becomes more homogeneous across the interface, and the effective barrier height progressively increases before reaching saturation [40].

This explains why all the curves show a rise in $\Phi_{Beffe\;}$with temperature**.** This result is in rigorous agreement with the experimental I–V–T characterization of Mo/4H–SiC Schottky diodes reported by Ouennoughi et al [38].

Their work confirms that for inhomogeneous contacts, $\Phi_{Beffe\;}$increases with temperature due to the Gaussian distribution of barrier heights, where carriers preferentially bypass higher potential regions at lower temperatures.

The fact that our analytical simulations perfectly replicate this experimentally observed behavior.

In Werner’s model, SB inhomogeneity parameter σplays a key role, as it reflects the degree of fluctuation in barrier heights across the metal/semiconductor interface.

**Fig 7:**
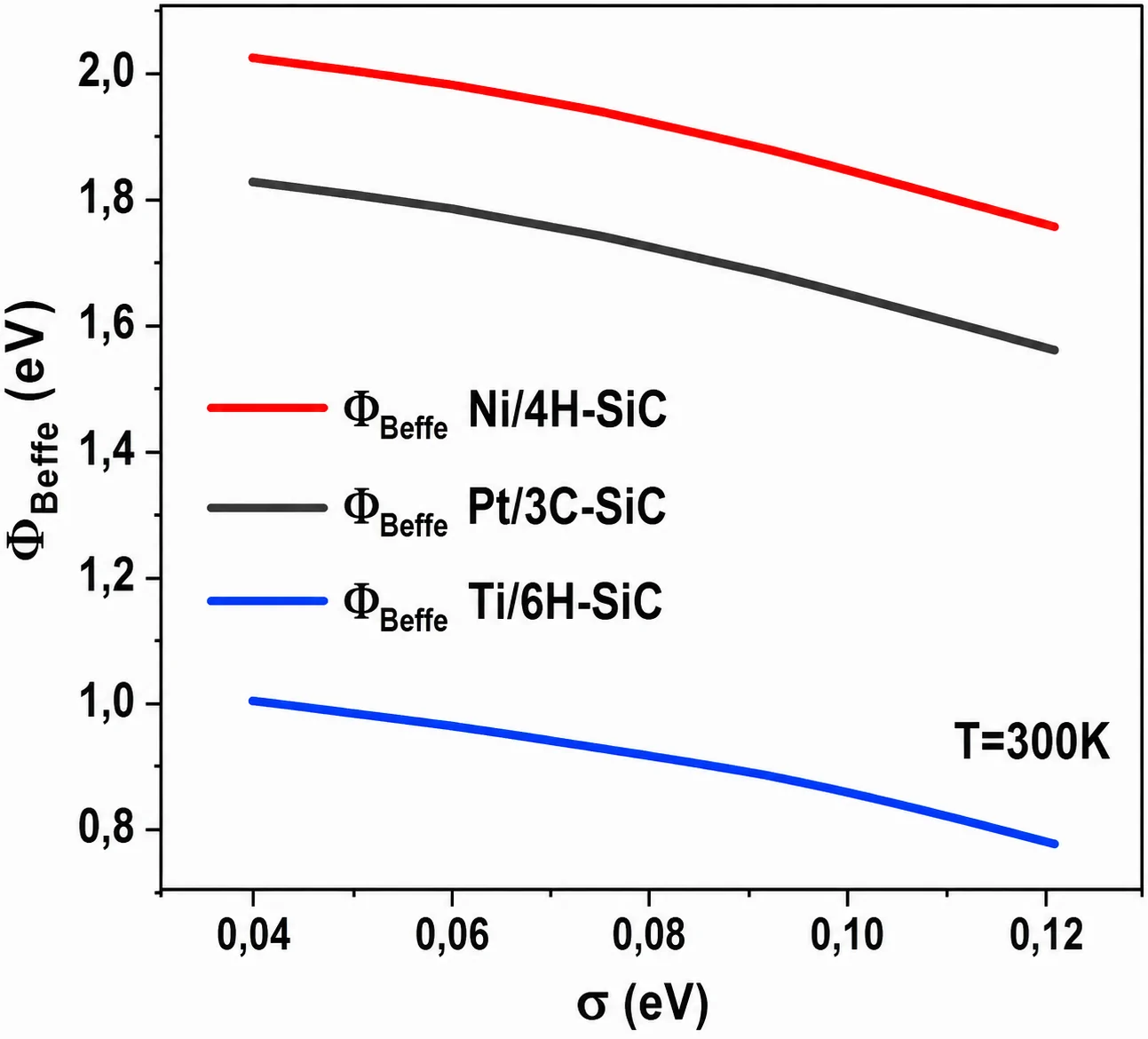
Variation of the Schottky barrier height as a function of standard deviation for Pt/3C-SiC, Ni/4H-SiC, and Ti/6H-SiC structures based on Werner model.

The Fig 7 shows the evolution of the effective SBH $\Phi_{Beffe\;}$ as a function of the standard deviation σ, at room temperature. As expected, all three structures exhibit a progressive decrease in $\Phi_{Beffe\;}$ as σ increases.

This behavior is due to the fact that a broader distribution of local barrier heights amplifies the influence of the lower-barrier regions in charge transport [7].

Electrons naturally follow the paths with the lowest energy barriers, so a more disordered interface leads to a lower effective barrier, even if the average barrier height remains unchanged [40].

These observations highlight the importance of controlling interface defects to ensure reproducibility and reliable performance of SiC-based Schottky devices.

#### 3.1.3. Tung SB.

In this section, we investigate the effect of temperature on the effective barrier height described by the Tung model, as expressed in equation (13).

The inhomogeneity parameter was set to a reference value of γ = 4 × 10^;⁻4^ cm^2/3^V^1/3^. This specific choice is physically justified by the foundational work of Tung et al.[19], where this value represents the upper boundary used to model localized barrier patches and simulate non-ideal Schottky current-voltage characteristics.

Furthermore, this value sits perfectly within the experimental range of 2 × 10 ⁻ ^3^ to 7 × 10^;⁻4^ cm^2/3^ V^1/3^ extracted by Omar et al. for Ni-Si/4H-SiC diodes, making it a highly representative benchmark for evaluating localized potential fluctuations in practical SiC devices [8,20].

On a microscopic scale, Tung’s patch model provides a rigorous interpretation of transport anomalies by considering discrete low-barrier regions embedded within a uniform high-barrier matrix.

The physical significance of the inhomogeneity parameter γ lies in its ability to dictate the electrostatic pinch-off effect at the interface.

When the γ increases representing either a larger patch radius or a greater potential departure the lateral electric field originating from the surrounding high-barrier regions strongly interacts with the potential profile of the low-barrier patches [41].

**Fig 8:**
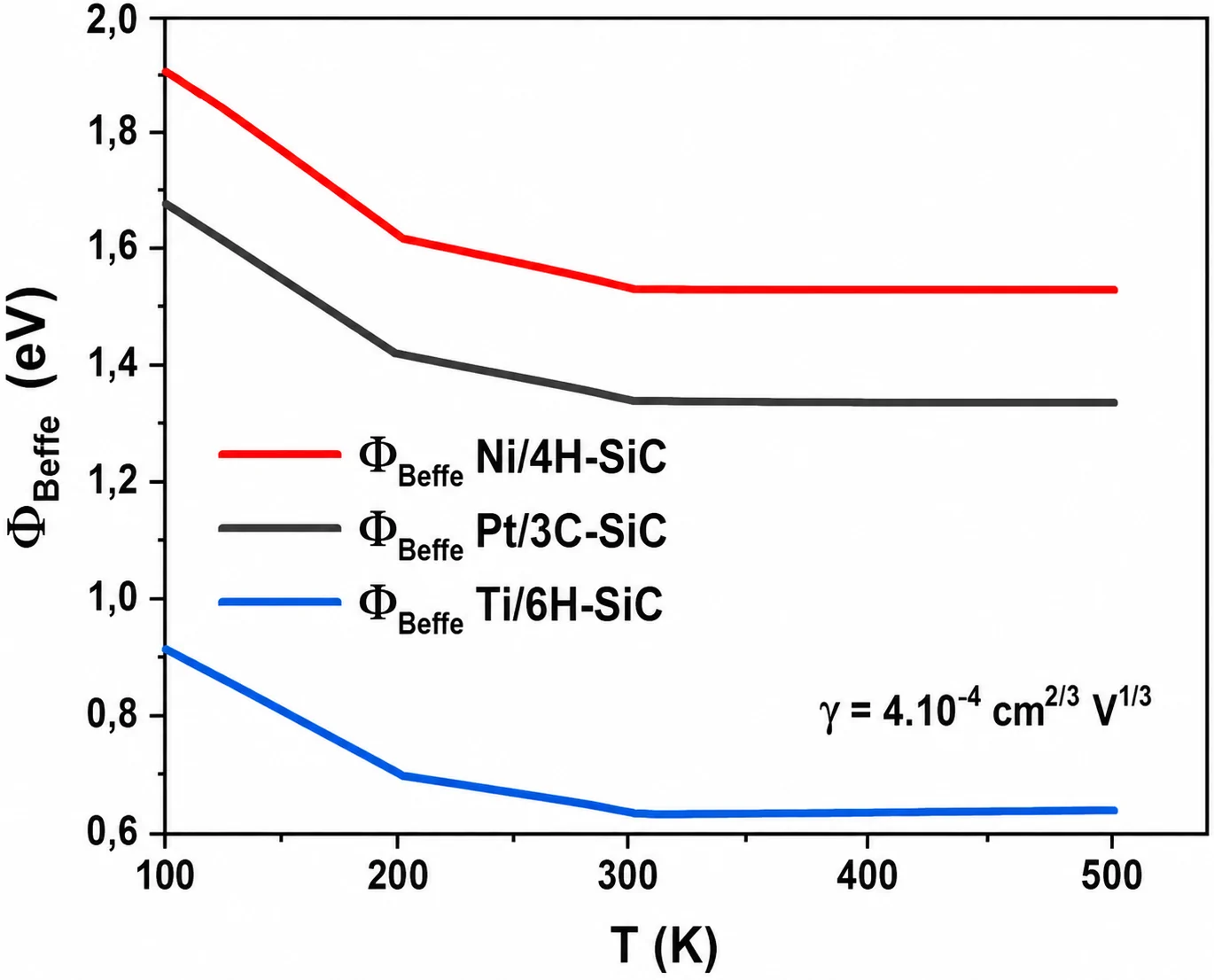
Variation of the Schottky barrier height as a function of temperature for Pt/3C-SiC, Ni/4H-SiC, and Ti/6H-SiC structures based on Tung’s model.

From Fig 8 the observed decrease in the effective SBH in Tung’s model for diodes based on the three SiC polytypes represents an intrinsic behavior linked to the transport mechanism assumed by this model.

At these moderate temperatures, current does not flow uniformly across the interface but is instead strongly channeled through local low-barrier regions, commonly referred to as patches.

At 100 K, electrons possess limited thermal energy. Consequently, current transport occurs mainly through localized low-barrier patches that remain energetically accessible. However, only a limited number of conduction paths contribute to the current at this temperature [5].

As temperature increases toward 300 K, two main effects occur [5]:

1. The number of thermally activated low-barrier patches increases, enabling additional localized conduction paths.
2. Current transport remains dominated by these low-barrier regions, while high-barrier regions contribute only weakly.

In this intermediate regime, the increasing contribution of low-barrier patches enhances localized transport and progressively lowers the extracted effective barrier height.

Beyond 300 K, the effective SB height tends to stabilize because the majority of the transport paths including those passing through higher-barrier regions become thermally accessible.

As a result, the current flow is no longer confined to low-barrier patches but starts to sample the entire inhomogeneous interface.

This leads to a saturation effect, where the measured barrier height reflects a statistical average of the spatial barrier distribution.

At this stage, the influence of local fluctuations diminishes, and the barrier becomes effectively temperature-independent within the model’s assumptions.

This result is in excellent agreement with the experimental study of Omar et al. [20] on Ni-Si/4H-SiC diodes. According to their findings, the pinch-off effect limits the current flow through these patches, making the effective barrier height extremely sensitive to thermal energy.

Our MATLAB simulations successfully replicate this phenomenon, showing that as temperature decreases, the current is increasingly ‘channeled’ through these inhomogeneities, a trend that perfectly matches the experimental extractions reported in the literature for SiC interfaces.

Another important factor in Tung’s model is the inhomogeneity parameter γ.

**Fig 9:**
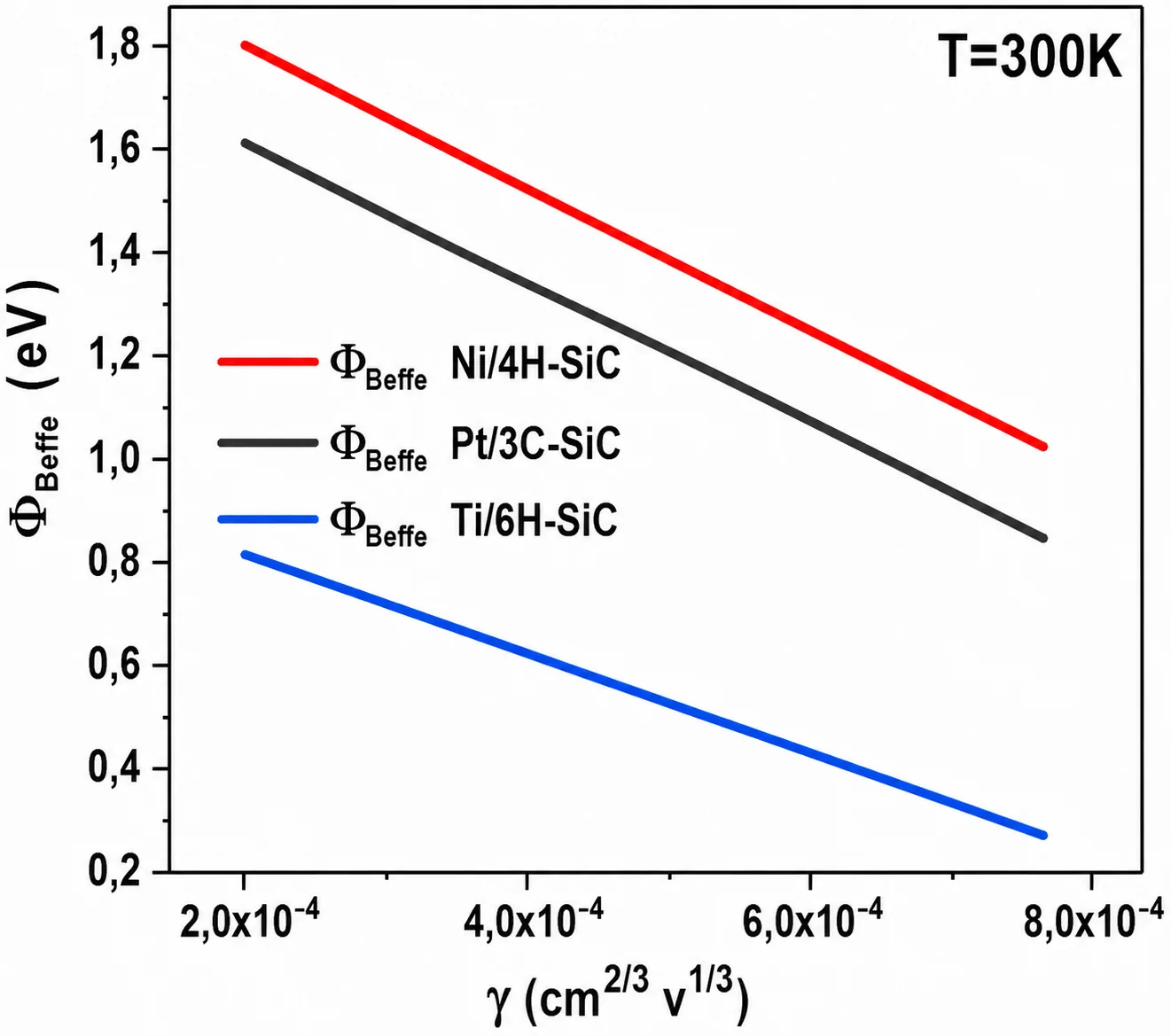
Variation of the Schottky barrier height as a function of inhomogenity parameter for Pt/3C-SiC, Ni/4H-SiC, and Ti/6H-SiC structures based on Tung’s model.

In Fig 9, the barrier height of the three Schottky diodes under investigation is plotted as a function of γ.

To investigate the influence of localized potential fluctuations within the framework of Tung’s model, the inhomogeneity parameter (γ) was analyzed across a range from 2×10^−4^ to 8× 10^−4^ cm^2/3^V^1/3^.

This selected range is physically justified by and closely aligned with the foundational literature on barrier height inhomogeneities.

Specifically, the seminal work of Tung. [19] evaluated I-V characteristics using gamma values ranging from 2×10^−4^ to 4× 10^−4^ cm^2/3^V^1/3^, while the comprehensive experimental study by Omar et al. [20] on Ni-Si/4H-SiC diodes extracted the Schottky barrier parameters and investigated interface non-uniformity for 2×10^−3^ to 7× 10^−4^ cm^2/3^V^1/3^.

The parameter γ characterizes the degree of lateral inhomogeneity at the interface. It reflects the density and size of local low-barrier patches.

For low γ values, these patches contribute weakly to the total current.

The barrier height observed experimentally remains relatively close to the ideal homogeneous value. However, as γincreases, the number or influence of these low-barrier patches grows, leading to two major physical effects [19]:

1. A larger fraction of the current is redirected through these locally favorable regions, as electrons naturally follow the paths of least resistance;
2. Transport becomes increasingly non-uniform across the interface, leading to a reduction in the apparent barrier height.

This phenomenon reflects a well-documented experimental reality. In real interfaces, once significant topographical or chemical irregularities are present, electrons tend to concentrate their flow through the most favorable microscopic paths. Increasing γ magnifies this current-funneling effect, allowing the influence of these *patches* to dominate, and thereby reducing the measured effective barrier height**.**

The consistently decreasing trend observed in the simulations confirms that even moderate increases in interfacial inhomogeneity can strongly distort the barrier profile experienced by charge carriers.

This simulated trend follows the pinch-off theory proposed by Tung [19] and is consistent with the experimental findings of Omar et al.[20], who reported that enhanced lateral inhomogeneities (larger γ) significantly lower the apparent average barrier height by redirecting a larger fraction of the current through locally favorable regions..

#### 3.1.4. Comparative temperature-dependent analysis.

To further emphasize the importance of temperature in Schottky barrier inhomogeneity, the thermal behavior predicted by the three investigated models is summarized in Table 2 and discussed below.

**Table 2:** Summary of the temperature dependence predicted by the three inhomogeneity models.

Model	Temperature dependence of effective SBH	Physical origin
Cowley and Sze	Slight increase for Pt/3C-SiC and Ni/4H-SiC; decrease for Ti/6H-SiC	Variation of interface-state pinning and bandgap
Werner	Monotonic increase	Reduced dominance of low-barrier regions
Tung	Decrease from 100 to 300 K, then saturation	Activation of localized low-barrier patches

Overall, the comparative analysis confirms that temperature plays a fundamental role in all three inhomogeneity models.

Although the specific thermal trends differ depending on the underlying physical mechanism, the simulations consistently demonstrate that temperature strongly affects both the effective Schottky barrier height and the resulting current–voltage characteristics.

These results highlight the necessity of incorporating temperature-dependent analysis when modeling SiC-based Schottky contacts intended for high-temperature power applications.

### 3.2. Study of the I–V characteristic for each model

In this section, the I–V characteristics are investigated with respect to the key parameters of each inhomogeneity model, for the Cowley and Sze model, the effect of interface states is studied by varying the interface state density (Nₛₛ).

In the case of the Werner model, the current–voltage behavior is analyzed as a function of the standard deviation (σ) representing the barrier height distribution.

Finally, for the Tung model, the inhomogeneity parameter (γ), which reflects the fraction of the contact area with lower barrier height, is considered.

This comparative analysis allows for a better understanding of how each parameter influences charge transport through the metal/semiconductor interface.

**Fig 10:**
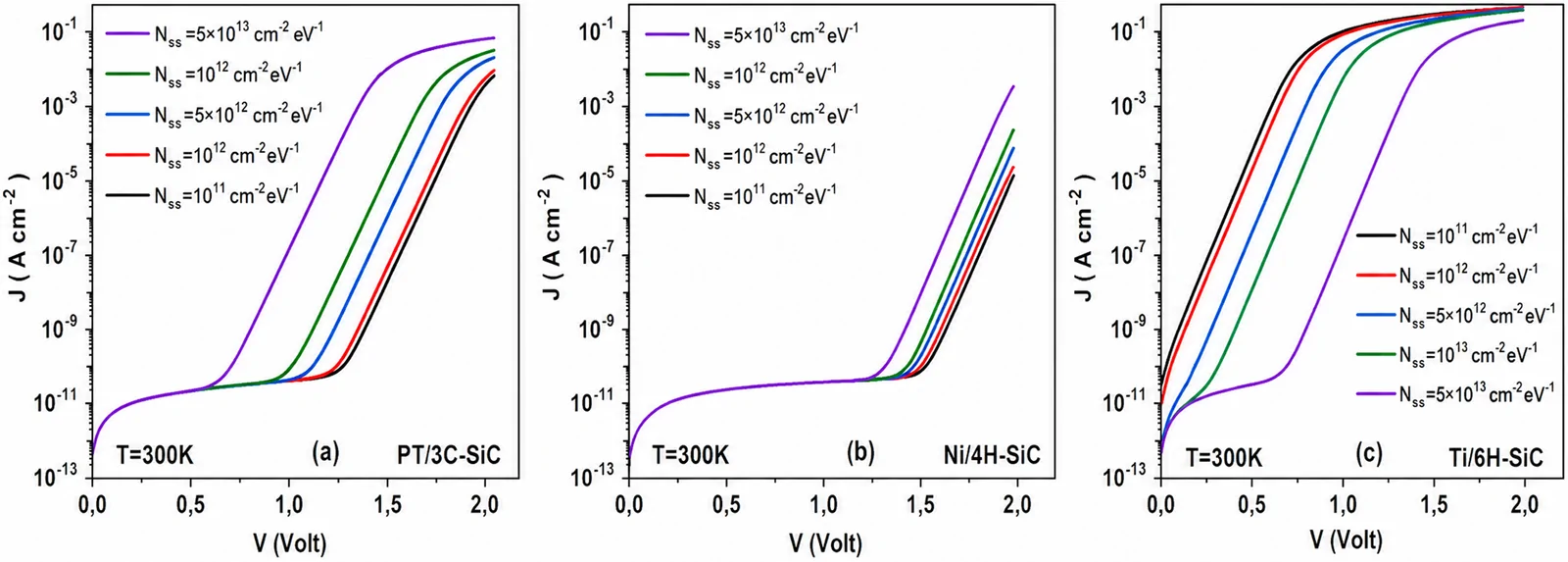
Effect of interface state density (Nₛₛ) on the I–V characteristics of (a) Pt/3C-SiC, (b) Ni/4H-SiC, and (c) Ti/6H-SiC Schottky diodes at T = 300 K based on the Cowley and Sze model.

From Fig 10, it can be seen that, for the 3C-SiC and 4H-SiC polytypes, increasing the interface state density N_ss_ leads to a reduction in the effective barrier height, which in turn results in higher forward currents in the I–V characteristics.

In contrast, for the 6H-SiC polytype, the effective barrier height increases as N_ss_ rises, and the corresponding I–V curves exhibit a clear decrease in forward current with increasing N_ss_.

To verify the accuracy of the implemented analytical model, the simulated forward current-voltage (I-V) characteristics at room temperature (T = 300 K) were compared with experimental results from the literature.

As shown in Fig 10, the simulated curve for the 4H-SiC polytype exhibits a high degree of similarity in terms of slope and current magnitude with the experimental I-V data reported by Bluet et al. [24] for high-voltage (4.5 kV) Ni/4H-SiC Schottky diodes.

**Fig 11:**
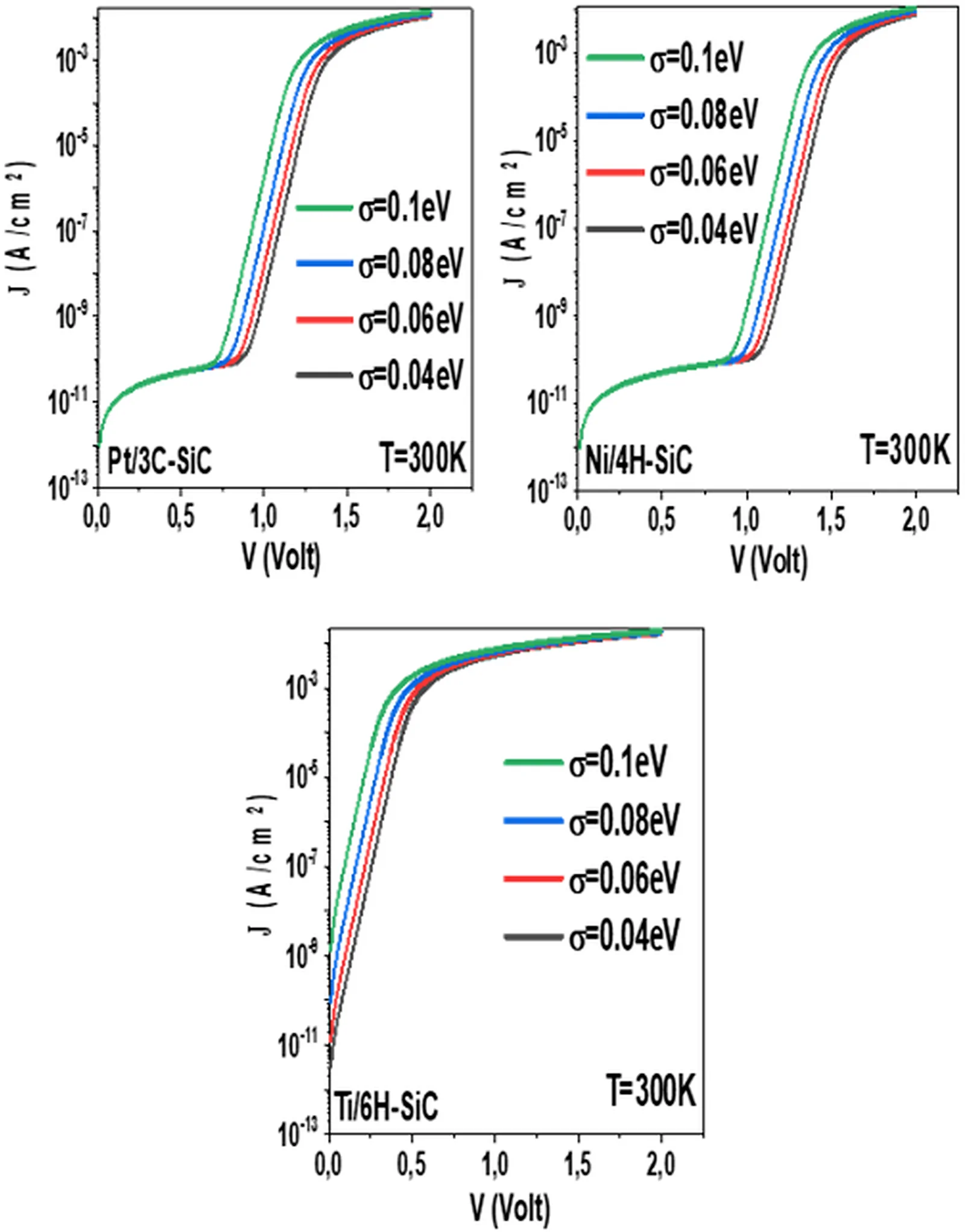
Simulated I–V characteristics based on Werner’s model for Pt/3C-SiC, Ni/4H-SiC, and Ti/6H-SiC diodes at various σ values.

The simulated current–voltage (I–V) characteristics for the three SiC-based Schottky diodes (Pt/3C-SiC, Ni/4H-SiC, and Ti/6H-SiC) at room temperature are presented in Fig 11 as a function of the standard deviation σ of the SBH distribution.

According to the Werner model, increasing σresults in a broader Gaussian distribution, which increases the likelihood of charge carriers encountering locally lower barrier regions.

This leads to a noticeable enhancement of the forward current, especially at lower voltages, where these inhomogeneities dominate the conduction.

The effect is more prominent in the Ti/6H-SiC diode, which already has the lowest mean barrier height, making it more sensitive to the tail of the distribution.

Conversely, Pt/3C-SiC and Ni/4H-SiC diodes show a relatively more moderate current increase with σ, consistent with their higher barrier heights.

These trends confirm the key role of σin determining the diode’s forward conduction behavior and illustrate the strength of the Werner model in capturing the impact of barrier inhomogeneities.

Latreche et al. [42] demonstrated, through an experimental study involving the extraction of the standard deviation σ at different temperatures, that σ increases with temperature, in a trend similar to that of the effective SBH.

These experimental findings reinforce the relevance of our simulated I-V characteristics versus σ, showing that the effect of Gaussian broadening is consistent with real behavior observed in the literature.

**Fig 12:**
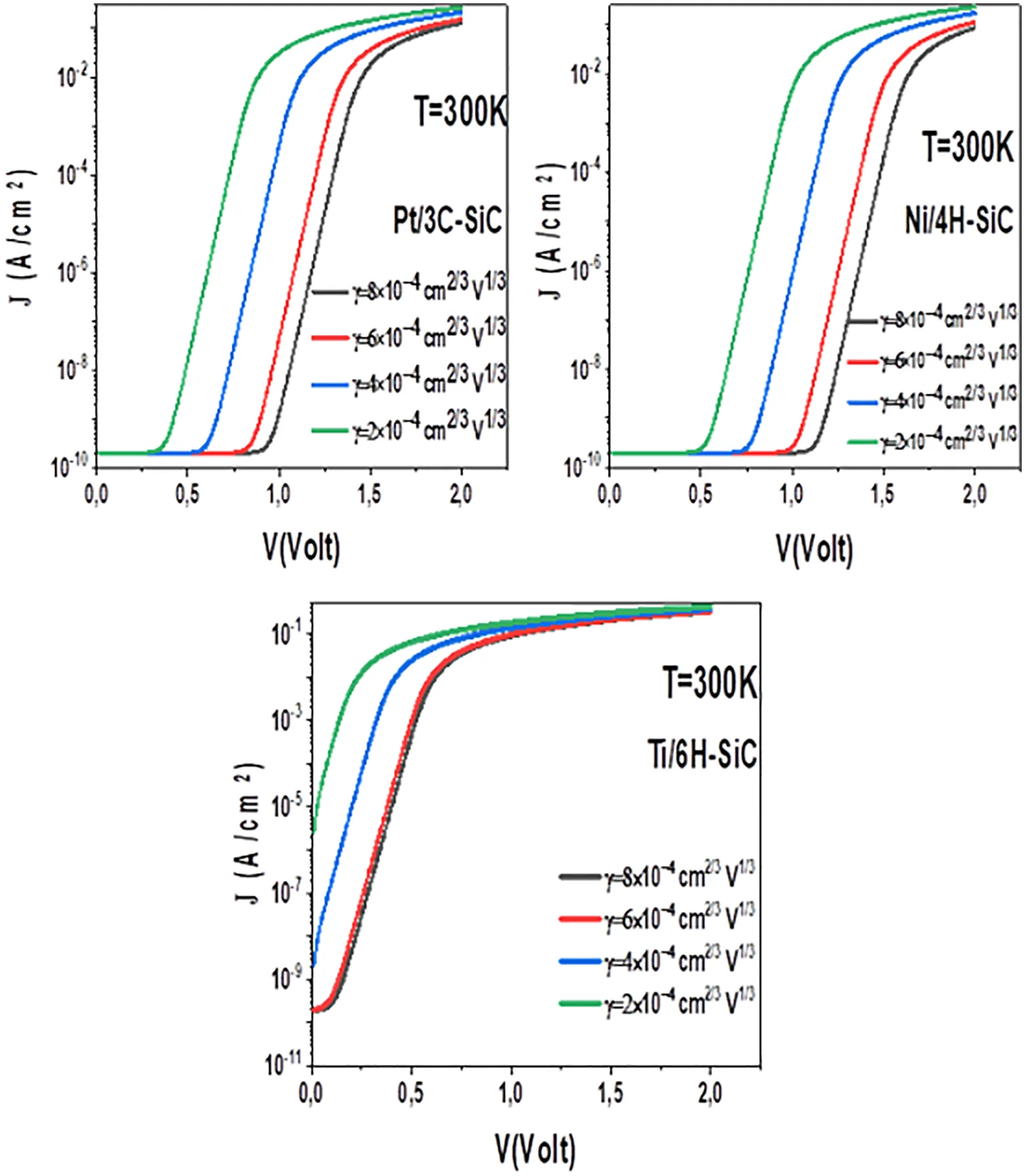
Simulated I–V characteristics based on Tung’s model for Pt/3C-SiC, Ni/4H-SiC, and Ti/6H-SiC diodes at various γ values.

The Fig 12 shows the simulated I -V characteristics based on the Tung model for three inhomogeneous Schottky diodes using different SiC polytypes: Pt/3C-SiC, Ni/4H-SiC, and Ti/6H-SiC.

The effect of the inhomogeneity parameter γ is analyzed by varying it from 2 × 10^−4^ to 8 × 10^−4^ cm^2/3^ v^1/3^.

A clear trend is observed: as γ decreases, the current increases significantly for a given forward bias. This behavior is attributed to stronger barrier inhomogeneities, which enhance transport through low-barrier patches.

In contrast, a higher γleads to a more uniform barrier and current behavior closer to the ideal thermionic emission.

This effect is especially pronounced in the forward conduction regime.

### 3.3. Comparison between the three models

In order to better understand the impact of interface inhomogeneities at the metal/SiC junction, this section provides a comprehensive comparison between the three most commonly used theoretical models: the Cowley and Sze model, the Werner model, and the Tung model.

For each SiC polytype (3C, 4H, and 6H), we simulated the I–V characteristics at 100 K, 300 K, and 500 K. These simulations highlight how the electrical behavior evolves with temperature, and allow for a direct comparison of the physical assumptions embedded in each model.

Three graphs are presented for each polytype, each showing the results of the three models at a given temperature. This visual approach helps to identify differences in behavior, particularly at low temperatures where the sensitivity to interface states and potential fluctuations becomes more pronounced.

In this section dedicated to comparing the I-V characteristics of the three models, the specific parameters for each model are:

- For the Cowley and Sze model, the interfacial oxide layer (d_ox_) is fixed at 20 Å, and the values of Nss are those listed in the Table 1.
- For the Werner model, the standard deviation sigma used in the simulation is equal to 0.09eV.
- For the Tunger model, the inhomogeneity parameter used in the simulation is equal to 4.10^−4^ cm^2/3^ V^1/3^.

**Fig 13:**
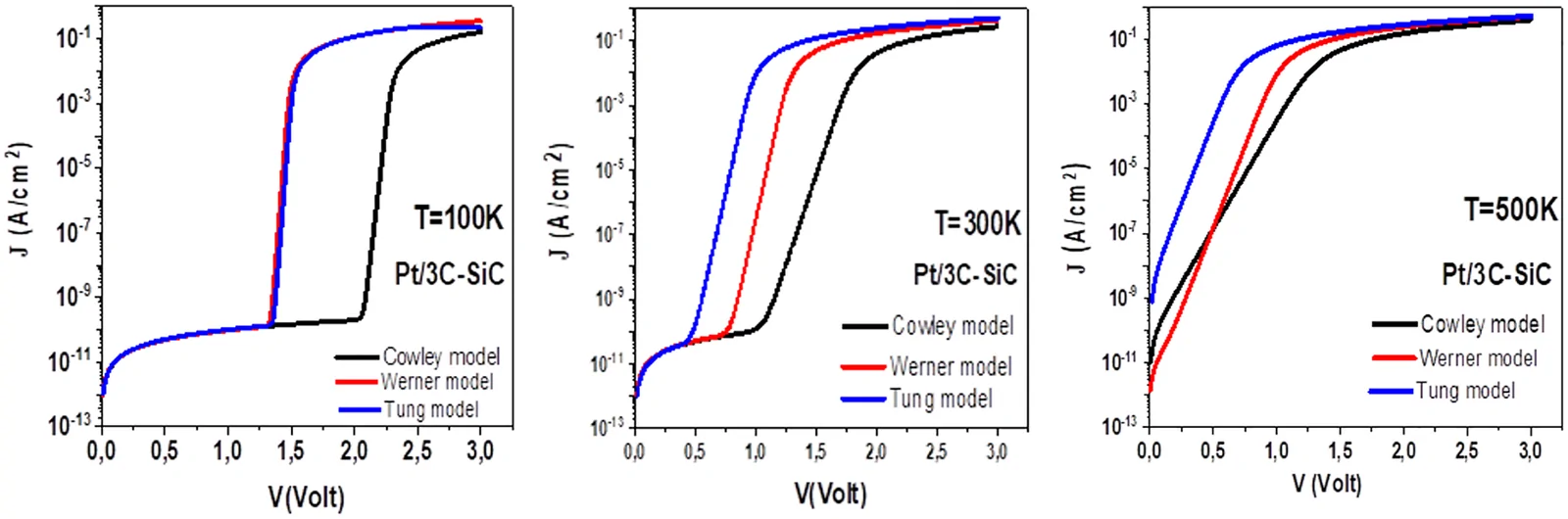
I-V characteristics of the Pt/3C-SiC Schottky diode at 100,300 and 500K simulated using the Cowley and Sze, Werner, and Tung inhomogeneity models.

**Fig 14:**
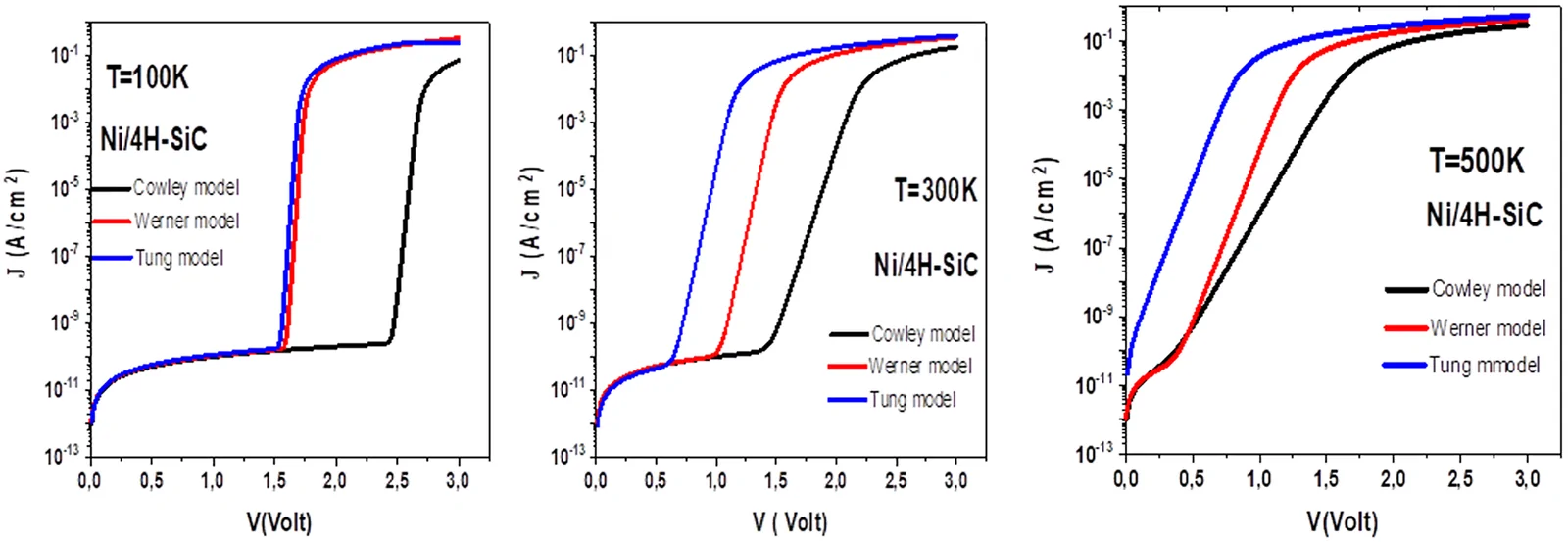
I-V characteristics of the Ni/4H-SiC Schottky diode at 100,300 and 500 K simulated using the Cowley and Sze, Werner, and Tung inhomogeneity models.

**Fig 15:**
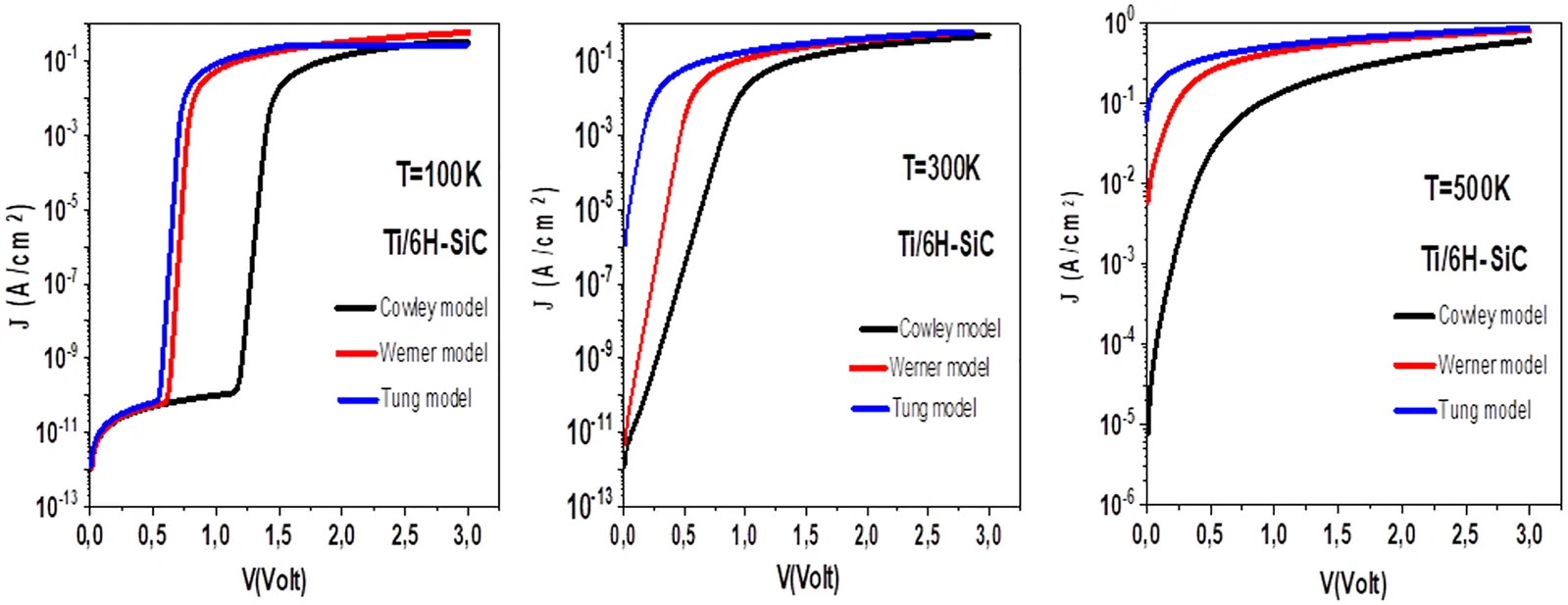
I-V characteristics of the Ti/6H-SiC Schottky diode at 100,300,500 K, simulated using the Cowley and Sze, Werner, and Tung inhomogeneity.

Figs 13–15 show the simulated I–V characteristics of Pt/3C-SiC, Ni/4H-SiC, and Ti/6H-SiC Schottky diodes at 100, 300, and 500 K.

Across all devices, increasing temperature shifts the curves toward higher currents at the same applied voltage, consistent with the thermionic emission mechanism.

The Cowley and Sze model exhibits the smallest variation in current with temperature, as the effective barrier remains relatively stable due to strong pinning.

A high interface state density thus limits the current and attenuates its thermal evolution. The Werner model reveals a more pronounced temperature dependence: as temperature increases, carriers access a broader portion of the inhomogeneous barrier landscape, low-barrier regions become less dominant, and the effective barrier approaches its average value, leading to a significant increase in forward current.

The Tung model shows the most pronounced deviation from ideal behavior at low temperature: even at 100 K, current is already significant due to conduction through locally depleted barrier regions. Between 100 and 300 K, this effect intensifies, then tends to stabilize around 500 K as transport gradually becomes more homogeneous across the interface.

At identical temperatures and for the same model, the relative order of the currents follows the expected hierarchy of average barriers: the Ti/6H-SiC diode exhibits the highest conduction, Ni/4H-SiC an intermediate level, and Pt/3C-SiC the lowest. However, the separation between these curves clearly depends on the considered inhomogeneity mechanism (pinning, statistical dispersion, or patch conduction).

Overall, the results demonstrate that interface physics plays a more important role in forward conduction than the crystallographic structure of the SiC polytype.

Interface state pinning (Cowley and Sze model) limits current, statistical barrier dispersion (Werner model) redistributes it toward lower values as temperature increases, while localized patch conduction (Tung model) amplifies it at low and intermediate temperatures.

Table 3 presents a comparison of the forward current density values for the three inhomogeneity models.

**Table 3:** Quantitative comparison of forward current density at 1.5 V for the three inhomogeneity models and SiC polytypes at different temperatures.

Model	Polytype	Forward Current density at 1.5 V and 100K (A /cm^2^)	Forward Current density at 1.5 V and 300K (A /cm^2^)	Forward Current density at 1.5 V and 500K (A /cm^2^)
Cowley–Sze	3C-SiC	1,8×10^−10^	7×10^−6^	4×10^−2^
Cowley–Sze	4H-SiC	1,6×10^−10^	6×10^−10^	4×10^−2^
Cowley–Sze	6H-SiC	2×10^−2^	11×10^−2^	24×10^−2^
Werner	3C-SiC	1×10^−3^	4×10^−2^	0,16
Werner	4H-SiC	1,53×10^−10^	3×10^−3^	4×10^−2^
Werner	6H-SiC	19×10^−2^	2×10^−1^	54×10^−1^
Tung	3C-SiC	1×10^−3^	4×10^−2^	0,18
Tung	4H-SiC	1,6×10^−10^	7×10^−3^	4×10^−2^
Tung	6H-SiC	26×10^−2^	32×10^−2^	5×10^−1^

Table 3 presents a quantitative comparison of the forward current density for the three SiC polytypes at different temperatures and for the three inhomogeneity models. Although significant differences in current magnitude are observed between polytypes, all models consistently predict thermally activated carrier transport, characterized by an overall increase in current density with temperature.

The results also indicate that the choice of the inhomogeneity model has a stronger influence on the predicted electrical characteristics than the SiC polytype itself.

### 3.4. Pratical device implications

The present modeling results provide several practical insights for the design and optimization of Schottky contacts in high-temperature silicon carbide (SiC) power devices.

In applications such as Schottky barrier diodes (SBDs), metal-semiconductor field-effect transistors (MESFETs), and junction barrier Schottky (JBS) diodes, the choice of the transport model significantly affects the predicted barrier height and current conduction behavior, which directly influences leakage current, forward voltage drop, and thermal stability [43].

Models that account for image-force lowering and interfacial effects, such as the Cowley–Sze and Padovani–Stratton formulations [6,44], generally predict lower effective barrier heights than the ideal thermionic emission model, resulting in higher reverse leakage currents.

This is particularly important for high-temperature operation, where leakage current can increase substantially and compromise device efficiency and long-term reliability [45–50].

The results also indicate that the impact of the transport model is often greater than the influence of the SiC polytype itself, emphasizing the importance of accurate physical modeling during contact engineering. From a practical standpoint, selecting appropriate contact metals, minimizing interface states, and controlling surface preparation are essential to achieve stable barrier heights and reduced current dispersion [14,51–56].

These considerations are critical for the development of robust SiC power devices intended for harsh environments such as electric vehicles, aerospace systems, and high-voltage energy conversion [46].

## 4. Conclusion

In summary, this work presented a comparative investigation of Schottky barrier inhomogeneity in 3C-, 4H-, and 6H-SiC using the Cowley–Sze, Werner, and Tung models under identical simulation conditions.

The results demonstrated that each model describes a distinct physical mechanism governing carrier transport at the metal/semiconductor interface, including interface-state pinning, statistical barrier-height fluctuations, and localized low-barrier patches.

Although quantitative differences were observed between the investigated SiC polytypes, all models consistently predicted thermally activated transport behavior and highlighted the strong influence of interface inhomogeneities on the effective Schottky barrier height and the corresponding I–V characteristics.

The comparative analysis further showed that the choice of the inhomogeneity model has a stronger impact on the predicted electrical behavior than the SiC polytype itself.

From an engineering perspective, reducing interface-state density, minimizing barrier-height fluctuations, and suppressing localized low-barrier patches are essential for improving barrier stability, reducing leakage current, and enhancing the thermal reliability of SiC Schottky power devices.

Overall, this study provides useful insights into the role of interface inhomogeneities and contributes to a better understanding of charge transport mechanisms in SiC-based Schottky contacts.

## Supporting information

S1 File MATLAB simulation code. (RAR)
