# Supplementary material for: Comparison of inhomogeneous Schottky barrier modeling approaches in SiC polytypes
Source: PLoS One. 2026 Aug 21;21(8):e0355480. doi: 10.1371/journal.pone.0355480 (PMC13496421; doi:10.1371/journal.pone.0355480)
Supplement: Supplementary file 1 [file pone.0355480.s002.docx]

**Reviewer Comments**

**Lack of Experimental Validation**

The study is entirely based on analytical simulations performed in MATLAB without any experimental verification. While the theoretical comparison is valuable, the absence of experimental data significantly limits the practical relevance of the findings. The authors are encouraged to validate their results with published experimental data or provide a critical comparison with literature-reported values.

**Insufficient Justification of Model Parameters**

Key parameters such as interface state density (Nss), standard deviation (σ), and inhomogeneity parameter (γ) are central to the analysis. However, the manuscript does not clearly justify the selected parameter ranges or their physical relevance to real SiC devices. A more detailed explanation or literature support is necessary.

**Limited Novelty in Model Comparison**

The three models (Cowley and Sze, Werner, and Tung) are well-established in the literature. The manuscript should more clearly highlight the novelty of this comparative study, particularly how it advances beyond existing reviews or prior comparative works.

**Inadequate Physical Interpretation**

Although the models are described, the discussion lacks depth in explaining the underlying physics of the observed trends. The manuscript would benefit from a more rigorous interpretation of how each parameter influences carrier transport and barrier formation in different SiC polytypes.

**Absence of Temperature-Dependent Analysis**

Schottky barrier inhomogeneity is strongly temperature dependent, especially in SiC-based power devices. The manuscript does not consider temperature effects, which is a significant limitation. Including temperature-dependent simulations would greatly strengthen the study.

**Comparison Between SiC Polytypes Needs Strengthening**

The conclusion that all three SiC polytypes show similar trends is not sufficiently supported with detailed quantitative comparison. The authors should provide clearer comparative plots, tabulated results, or normalized analysis to substantiate this claim.

**Practical Device Implications Are Underdeveloped**

While the manuscript mentions applications in high-temperature power devices, the discussion does not adequately connect the modeling results to real device design considerations (e.g., contact engineering, reliability, leakage current control). This aspect should be expanded.

**Clarity in MATLAB Simulation Methodology**

The manuscript lacks sufficient detail regarding the simulation procedure, boundary conditions, and assumptions used in MATLAB. Reproducibility requires a clearer description of equations, numerical methods, and convergence criteria.

**Graphical and Data Presentation**

The effectiveness of the comparative analysis depends heavily on visual representation. The manuscript should include clearer, high-quality plots showing I–V characteristics, SBH variation, and parameter sensitivity across models and polytypes.

**Language and Technical Presentation**

The manuscript contains several long and complex sentences that affect readability. Careful language editing is required to improve clarity, precision, and flow of the discussion.

**Recommendation: Major Revision**

The manuscript addresses an important topic and presents a useful comparative framework. However, substantial improvements are required in terms of validation, depth of analysis, methodological clarity, and practical relevance before it can be considered for publication.
