# Supplementary material for: Comparison of inhomogeneous Schottky barrier modeling approaches in SiC polytypes
Source: PLoS One. 2026 Aug 21;21(8):e0355480. doi: 10.1371/journal.pone.0355480 (PMC13496421; doi:10.1371/journal.pone.0355480)
Supplement: Supplementary file 2 [file pone.0355480.s004.docx]

**Manuscript Number: PONE-D-26-11447**

**Response to Reviewers**

We would like to thank the Editor and the Reviewers for their valuable time and constructive comments regarding our manuscript entitled **“Comparison of Inhomogeneous Schottky Barrier Modeling Approaches in SiC Polytypes”** submitted to *PLOS ONE*. We sincerely appreciate the insightful suggestions and comments provided by the Reviewers, which helped us improve the quality and clarity of the manuscript. We have carefully addressed all comments and incorporated the corresponding revisions in the updated version of the paper.All modifications made in the revised manuscript are highlighted in **red**. A detailed point-by-point response to each reviewer comment is provided below in **blue**.

**1.Lack of Experimental Validation**

The study is entirely based on analytical simulations performed in MATLAB without any experimental verification. While the theoretical comparison is valuable, the absence of experimental data significantly limits the practical relevance of the findings. The authors are encouraged to validate their results with published experimental data or provide a critical comparison with literature-reported values.

**Author reponse :**

We sincerely thank the reviewer for this valuable suggestion. We agree that comparing theoretical modeling with experimental results is important to demonstrate the physical relevance of our study. In the revised manuscript, we improved the discussion by directly comparing our simulation results with previously reported experimental studies as folluws :

- Cowley–Sze Validation (N_ss_): The simulated decrease in N_ss_ with increasing temperature has been cross-referenced with the experimental work of **Benamara et al. [29]** on SiC Schottky diodes and the TCAD simulations of **Toumi et al. [30]** on GaAs structures, both confirming the thermal emission of trapped charges.
- Werner Model Validation ($\sigma$ Trends): Our simulated temperature dependence of the effective barrier height is in rigorous agreement with the experimental I-V-T characteristics reported by **Ouennoughi et al [38]** on Mo/4H-SiC Schottky diodes.
- Tung Model Validation ($\gamma$): The simulated carrier-channeling at low temperatures (Figure 8) and the barrier lowering via the "pinch-off" effect (Figure 9) accurately replicate the experimental extractions of ***Omar et al. [20]*** on Ni-Si/4H-SiC diodes, confirming the validity of our local potential fluctuation modeling.
- Current-Voltage (I-V) Validation: To secure a final layer of quantitative validation, the room-temperature forward I-V characteristics simulated in Figure 10 show excellent visual and quantitative slope alignment with the experimental data from high-voltage (4.5 kV) Ni/4H-SiC diodes published by ***Bluet et al[24]****.*

By aligning our MATLAB frameworks with these diverse experimental and numerical studies, we ensure that our comparative analysis accurately reflects the physical transport dynamics of real-world SiC devices.

**Changes in the Manuscript:**

Sections 3.1.1, 3.1.2, 3.1.3 (Pages 11,15 and 20 ): New comparative discussions have been integrated to explicitly link Figures 3, 6 and 8 with the experimental data of *Benamara et al.[29] Ouennoughi et al[38] and* *Omar et al.[20]*

Section 3.3 (Page 23): A dedicated text insertion has been made to highlight the quantitative agreement between Figure 10 and the experimental data of *Bluet et al[24]****.***

**2-Insufficient Justification of Model Parameters**

Key parameters such as interface state density (Nss), standard deviation (σ), and inhomogeneity parameter (γ) are central to the analysis. However, the manuscript does not clearly justify the selected parameter ranges or their physical relevance to real SiC devices. A more detailed explanation or literature support is necessary.

**Author reponse :**

We thank the reviewer for highlighting the need for a clearer justification of our parameters.

**2.1** **Justification of the standard deviation value (σ)**

In the revised manuscript, we have set the standard deviation to σ **= 0.09eV**. This choice is not arbitrary but is based on a rigorous survey of experimental extractions from the literature for SiC Schottky diodes:

- **Ouennoughi et al.[38]** reported a value of **0.08 eV** for Mo/4H-SiC contacts.
- **Bluet et al.[24]** obtained **0.09 eV** for high-voltage Ni/4H-SiC diodes.
- **Toumi et al.[37]** extracted **0.092 eV** for similar SiC-based structures.

By selecting **0.09 eV** as a representative mean value, our model gains physical credibility and aligns perfectly with the current state of experimental research. We believe this firmly addresses the concern regarding the physical relevance of our simulation parameters.

**2.2.** **Justification of the Inhomogeneity Parameter Range (**$\boldsymbol{\gamma}$**):**

The selected range for the patch inhomogeneity parameter, 2$\times$10^-4^ to 8$\times$ 10^-4^ cm²ᐟ³V¹ᐟ³, is directly based on the values reported in foundational and experimental literature:

- **Tung et al. [19]:** analyzed the Schottky barrier inhomogeneities across a $\boldsymbol{\gamma}$ range of 2$\times$10^-4^ to 4$\times$ 10^-4^ cm²ᐟ³V¹ᐟ³.
- **Omar et al. [20]:** extracted the Schottky barrier parameters for $\boldsymbol{\gamma}$ values spanning from 2$\times$10^-3^ to 7$\times$ 10^-4^ cm²ᐟ³V¹ᐟ³ on Ni-Si/4H-SiC interfaces.

Our simulation range 2$\times$10^-4^ to 8$\times$ 10^-4^ cm²ᐟ³V¹ᐟ³ was established directly on this basis to cover these theoretical and experimental boundaries while accounting for slightly higher potential fluctuations.

**2.3 Physical Justification of the N_ss_ Values**

We agree with the reviewer that the range of the interface state density must reflect real device characteristics. To ensure strict physical relevance to the studied material, the selected range for our N_ss_ analysis (5$\times$10^11^ to 5$\times$10^13^ cm^-2^ eV^-1^) has been validated against the experimental work of **Khanna et al.[32]**, who specifically investigated metal/4H-SiC Schottky interfaces and **Benamara et al.[29].**

**Khanna et al**. reported extracted N_ss_ values in the range of 2$\times$10^12^ to 6.5$\times$10^12^ cm^-2^ eV^-1^. And **Benamara et al**., who worked on the Ni/6H-SiC diode, reported a reduction in the interface state density Nss from 1.2×10^13^ to 6×10^12^ cm−^2^eV^−1^. Our selected range directly encompasses these experimental values.

**Changes in the Manuscript:**

**Section 3.1.2 (page 15):** A new paragraph has been added to justify the choice of σ = 0.09 eV by referencing the experimental works of *Ouennoughi et al.[37], Bluet et al.[24], and Toumi et al [36].*

**Section 3.2 (page 19) :** The text has been updated to explicitly specify that our simulation range is built upon the respective ranges of *Tung et al.[19] and Omar et al.[20].*

**Section 3.1.1 (page 13) :** The text has been updated to explicitly justify the selected N_ss_ range by referencing the experimental boundaries established by *Khanna et al.[32]* for 4H-SiC structures and *Benamara et al. [29].*

**3-Limited Novelty in Model Comparison**

The three models (Cowley and Sze, Werner, and Tung) are well-established in the literature. The manuscript should more clearly highlight the novelty of this comparative study, particularly how it advances beyond existing reviews or prior comparative works.

**Author reponse:**

We completely understand the reviewer's concern regarding the well-established nature of the individual models. However, we would like to clarify that the novelty of this work does not lie in the development of new analytical formulations, but rather in the simultaneous quantitative benchmark and unified mapping of these frameworks applied to SiC polytypes (4H, 6H, and 3C). While existing literature typically reviews these models qualitatively or applies a single model to fit specific experimental data, our study advances prior works in three distinct ways:

1. **Unified Multi-Model Confrontation :** We implement a side-by-side quantitative comparison of the interface-state model (Cowley-Sze), the macroscopic fluctuation model (Werner), and the microscopic patch model (Tung) under identical simulation boundaries. This directly identifies the exact temperature and voltage thresholds where each model loses its physical validity.
2. **Polytype-Specific Sensitivity Mapping :** We systematically correlate the variation of inhomogeneity parameters (σ, $\gamma$ and N_ss_) with the physical properties of different SiC polytypes, providing a clear reference grid for device designers to know which model is most accurate depending on the specific polytype and defect density.
3. **Transition Dynamics :** Our framework clearly demonstrates how microscopic potential patches (Tung) macroscopically average out into a Gaussian distribution (Werner) as a function of temperature, bridging the gap between these two theories in a way that standard reviews do not quantify.

We have significantly revised the Introduction and Conclusion sections to explicitly highlight these contributions and clearly state how this study provides a predictive tool rather than a simple literature review

**Changes in the Manuscript:**

**Section 1 (Introduction, page 3) :** The paragraph preceding Figure 1. has been rewritten to explicitly frame the novelty, objectives, and distinct advantages of this comparative framework over existing literature.

**Section 4 (Conclusion, page 33) :** We have revised the conclusion to emphasize the practical engineering insights gained from this simultaneous multi-model evaluation.

**4-Inadequate Physical Interpretation**

Although the models are described, the discussion lacks depth in explaining the underlying physics of the observed trends. The manuscript would benefit from a more rigorous interpretation of how each parameter influences carrier transport and barrier formation in different SiC polytypes.

**Author reponse :**

We thank the reviewer for this insightful comment. We agree that strengthening the physical interpretation of the carrier transport mechanisms across the different SiC polytypes enhances the depth of the paper. In the revised manuscript, we have expanded the discussion sections to explicitly connect our simulation parameters (σ, $\gamma$ and N_ss_) with the intrinsic semiconductor physics of 4H, 6H, and 3C-SiC:

1. **Standard deviation (**σ)**:** We have clarified that the temperature dependence of the effective barrier height ($\boldsymbol{\Phi}_{\mathbf{B}}$) is governed by the competitive trade-off between thermal energy and the standard deviation (σ). At low temperatures, carriers lack sufficient energy to surmount the nominal barrier and are forced to flow through localized patches with lower potential energy. This effect is more pronounced in wider bandgap polytypes (4H-SiC, E_g_ = 3.26 eV) compared to lower bandgap polytypes (3C-SiC, E_g_ = 2.3 eV), as the relative magnitude of potential fluctuations impacts carrier distribution more severely when the ideal barrier height is larger [34][35] [36].
2. **inhomogeneity parameter** $\boldsymbol{(}\boldsymbol{\gamma}\boldsymbol{)}$**:** We have added a comprehensive explanation of the pinch-off effect dictated by the inhomogeneity parameter $\gamma$. When $\gamma$ increases, thelateral electric field arising from the surrounding high-barrier regions strongly interacts with the low-barrier patches. This electrostatic interaction modulates the potential profile, altering the effective transmission coefficient of the carriers and deviating the transport from the pure thermionic emission (TE) theory [8][20][41].
3. **Interface states (N_ss_):** We have enriched the text to discuss how the variations in N_ss_ across the polytypes directly influence the degree of Fermi-level pinning. Higher calculated N_ss_ values signify a greater screen of the metal work function by the interface traps, which severely limits the control of the barrier formation by the choice of the contact metal [6].

**Changes in the Manuscript:**

**Sections 3.1.1, 3.1.2, and 3.1.3 (Pages 11,14 ,18,19) :** Detailed paragraphs containing these physical interpretations and transport dynamics have been integrated to accompany each respective simulation curve.

**5-Absence of Temperature-Dependent Analysis**

Schottky barrier inhomogeneity is strongly temperature dependent, especially in SiC-based power devices. The manuscript does not consider temperature effects, which is a significant limitation. Including temperature-dependent simulations would greatly strengthen the study.

**Author reponse :**

We thank the reviewer for this valuable comment. To make the temperature-dependent analysis more explicit and easier to identify, a new section entitled “Comparative Temperature-Dependent Analysis” has been added in the revised manuscript. This section provides a dedicated discussion and a summary table highlighting the influence of temperature on the effective Schottky barrier height in the Cowley–Sze, Werner, and Tung models.In addition, we would like to clarify that temperature-dependent simulations were already included in the original manuscript. Specifically, Figures 3, 4, 6, and 8 present the variation of the interface state density and effective Schottky barrier height as a function of temperature, while Figures 13–15 show the corresponding I–V characteristics at 100 K, 300 K, and 500 K for all three SiC polytypes.

| **Model** | **Temperature dependence of effective SBH** | **Physical origin** |
| --- | --- | --- |
| Cowley and Sze | Slight increase for Pt/3C-SiC and Ni/4H-SiC; decrease for Ti/6H-SiC | Variation of interface-state pinning and bandgap |
| Werner | Monotonic increase | Reduced dominance of low-barrier regions |
| Tung | Decrease from 100 to 300 K, then saturation | Activation of localized low-barrier patches |

**Changes in the Manuscript:**

An additional section (Section 3.1.4, page 21) was dedicated to the role of temperature and its effect on the three inhomogeneous models, including the addition of a comparative table (Table 2).

**6-Comparison Between SiC Polytypes Needs Strengthening**

The conclusion that all three SiC polytypes show similar trends is not sufficiently supported with detailed quantitative comparison. The authors should provide clearer comparative plots, tabulated results, or normalized analysis to substantiate this claim.

**Author reponse**

We thank the reviewer for this valuable comment. To provide a clearer and more quantitative comparison, we have added **Table 3**, entitled “Quantitative Comparison of Forward Current Density at 1.5 V for the Three Inhomogeneity Models and SiC Polytypes at Different Temperatures.” This table summarizes the forward current density values at 1.5 V for all three SiC polytypes (3C-SiC, 4H-SiC, and 6H-SiC) at 100 K, 300 K, and 500 K. The added quantitative results show that, although significant differences exist in the absolute current-density values, all three SiC polytypes exhibit a consistent thermally activated behavior within each inhomogeneity model. In particular, the Werner and Tung models predict a progressive increase in forward current with temperature for all investigated polytypes. These results confirm that the predicted electrical behavior is influenced more strongly by the underlying inhomogeneity model than by the SiC polytype itself

| **Model** | **Polytype** | **Forward Current density at 1.5 V and 100K (A /cm^2^)** | **Forward Current density at 1.5 V and 300K (A /cm^2^)** | **Forward Current density at 1.5 V and 500K (A /cm^2^)** |
| --- | --- | --- | --- | --- |
| Cowley–Sze | 3C-SiC | 1,8$\times$10^-10^ | 7$\times$10^-6^ | 4$\times$10^-2^ |
| Cowley–Sze | 4H-SiC | 1,6$\times$10^-10^ | 6$\times$10^-10^ | 4$\times$10^-2^ |
| Cowley–Sze | 6H-SiC | 2$\times$10^-2^ | 11$\times$10^-2^ | 24$\times$10^-2^ |
| Werner | 3C-SiC | 1$\times$10^-3^ | 4$\times$10^-2^ | 0,16 |
| Werner | 4H-SiC | 1,53$\times$10^-10^ | 3$\times$10^-3^ | 4$\times$10^-2^ |
| Werner | 6H-SiC | 19$\times$10^-2^ | 2$\times$10^-1^ | 54$\times$10^-1^ |
| Tung | 3C-SiC | 1$\times$10^-3^ | 4$\times$10^-2^ | 0,18 |
| Tung | 4H-SiC | 1,6$\times$10^-10^ | 7$\times$10^-3^ | 4$\times$10^-2^ |
| Tung | 6H-SiC | 26$\times$10^-2^ | 32$\times$10^-2^ | 5$\times$10^-1^ |

**Changes in the Manuscript:**

**Sections 3.3 (Page 32) :** We have added Table 3, which presents a comparison of the forward current density values for the three inhomogeneity models.

**7-Practical Device Implications Are Underdeveloped**

While the manuscript mentions applications in high-temperature power devices, the discussion does not adequately connect the modeling results to real device design considerations (e.g., contact engineering, reliability, leakage current control). This aspect should be expanded.

**Author reponse :**

Thank you for this valuable comment. In response, we have added a new subsection entitled **“**Practical Device Implications**”** in the Discussion section, immediately before the Conclusion. This subsection expands the connection between the modeling results and practical design considerations for high-temperature SiC power devices.

The added discussion explains how the choice of transport model affects the predicted Schottky barrier height and current transport characteristics, and how these differences directly influence key device parameters such as reverse leakage current, forward voltage drop, thermal stability, and long-term reliability. We also discuss the implications for contact engineering, including metal selection, interface-state reduction, and surface preparation, as well as the relevance of these considerations to applications such as Schottky barrier diodes, JBS diodes, and MESFETs operating in harsh environments.

Relevant references have been added to support this discussion, including **L. M. Porter et al. [43]**, **Padovani and Stratton [44], and Bounab et al. [45].**

**Changes in the Manuscript:**

An additional section (Section 3.4, page 32,33) was dedicated to highlight how the modeling results can guide Schottky contact engineering and leakage current control in high-temperature SiC power devices.

**8-Clarity in MATLAB Simulation Methodology**

The manuscript lacks sufficient detail regarding the simulation procedure, boundary conditions, and assumptions used in MATLAB. Reproducibility requires a clearer description of equations, numerical methods, and convergence criteria.

**Author reponse**

We completely agree with the reviewer that technical transparency and reproducibility are vital for a simulation-based study. In accordance with this valuable recommendation, we have expanded the methodology description in the revised manuscript to provide a comprehensive, step-by-step disclosure of our MATLAB simulation framework.

To clarify the exact numerical procedure used in this work:

1. **Physical Assumptions & Equations:** The current-voltage (I-V) characteristics are modeled using the thermionic emission framework, which accounts for the simultaneous presence of the voltage-dependent effective barrier height and the series resistance (Rs). This yields an implicit non-linear transcendental equation where the actual junction voltage equals (Va - IRs).
2. **Numerical Method:** Since this implicit equation cannot be solved analytically, we implemented the **Newton-Raphson iterative method** in MATLAB to extract the exact forward current (I) for each applied bias step.
3. **Simulation Boundary Conditions & Steps:** The forward bias voltage (Va) was swept continuously from a boundary of **0 V to 3 V** using a precise voltage step size (Pas) of **0.01 V** to ensure high-resolution tracking of the diode's turning-on regime.
4. **Convergence Criteria:** To ensure numerical accuracy and physical consistency, the Newton–Raphson iterative procedure was continued until the difference between two successive current values became smaller than 10^−12^ A, corresponding to a relative error below 10^−6^. At this point, the solution was considered converged and the calculated current was taken as the final result

We have integrated these missing numerical details into a new structured paragraph within the revised manuscript to ensure full reproducibility.

**Changes in the manuscript**

**section 3 (pages 8,9)**: We have added a detailed description of the MATLAB simulation procedure.

**9-Graphical and Data Presentation**

The effectiveness of the comparative analysis depends heavily on visual representation. The manuscript should include clearer, high-quality plots showing I–V characteristics, SBH variation, and parameter sensitivity across models and polytypes.

Author reponse :

Thank you for this valuable comment. In the revised manuscript, we improved the quality and readability of all figures to enhance the visual presentation of the comparative analysis. All plots were regenerated at higher resolution (1200 pixels, 24-bit color), and the labels, legends, and plotted data were reformatted to ensure that all curves and annotations are clearly visible. These improvements significantly enhance the clarity of the I–V characteristics, Schottky barrier height variations, and parameter sensitivity plots.

**10-Language and Technical Presentation**

The manuscript contains several long and complex sentences that affect readability. Careful language editing is required to improve clarity, precision, and flow of the discussion.

Author answer :

We sincerely thank the reviewer for this valuable comment regarding the language and technical presentation of the manuscript. In response, the manuscript has been carefully revised to improve readability, clarity, and the overall flow of the discussion. Several long and complex sentences were rewritten into shorter and more concise formulations, particularly in the results and discussion section.

For example, in section 3.1.2 the sentence : “The current distribution becomes more uniform across the contact, and the effective barrier height increases, eventually saturating as electrons access the full range of interface inhomogeneities.”was simplified to: Consequently, current transport becomes more homogeneous across the interface, and the effective barrier height progressively increases before reaching saturation.

Similarly, the sentence: At 100 K, although electrons have very low thermal energy, they can still traverse these low-barrier patches either due to favorable energetic alignment or through thermally assisted tunneling.”was revised as: At 100 K, electrons possess limited thermal energy. Nevertheless, current can still flow through low-barrier patches due to favorable local energy alignment and thermally assisted tunneling.
