# Supplementary material for: Comparison of inhomogeneous Schottky barrier modeling approaches in SiC polytypes
Source: PLoS One. 2026 Aug 21;21(8):e0355480. doi: 10.1371/journal.pone.0355480 (PMC13496421; doi:10.1371/journal.pone.0355480)
Supplement: Peer review history

# Peer review: Decision Letter 0

**DOI:** 10.1371/journal.pone.0355480.r001

**Authors:** Latha Marasamy

12 May 2026

Dear Dr.  Habib,

Thank you for submitting your manuscript to PLOS ONE. After careful consideration, we feel that it has merit but does not fully meet PLOS ONE’s publication criteria as it currently stands. Therefore, we invite you to submit a revised version of the manuscript that addresses the points raised during the review process.

Please submit your revised manuscript by Jun 26 2026 11:59PM. If you will need more time than this to complete your revisions, please reply to this message or contact the journal office at plosone@plos.org. When you're ready to submit your revision, log on to https://www.editorialmanager.com/pone/ and select the 'Submissions Needing Revision' folder to locate your manuscript file.

- A letter that responds to each point raised by the academic editor and reviewer(s). You should upload this letter as a separate file labeled 'Response to Reviewers'.
- A marked-up copy of your manuscript that highlights changes made to the original version. You should upload this as a separate file labeled 'Revised Manuscript with Track Changes'.
- An unmarked version of your revised paper without tracked changes. You should upload this as a separate file labeled 'Manuscript'.

If you would like to make changes to your financial disclosure, please include your updated statement in your cover letter. Guidelines for resubmitting your figure files are available below the reviewer comments at the end of this letter.

If applicable, we recommend that you deposit your laboratory protocols in protocols.io to enhance the reproducibility of your results. Protocols.io assigns your protocol its own identifier (DOI) so that it can be cited independently in the future. For instructions see: https://journals.plos.org/plosone/s/submission-guidelines#loc-laboratory-protocols. Additionally, PLOS ONE offers an option for publishing peer-reviewed Lab Protocol articles, which describe protocols hosted on protocols.io. Read more information on sharing protocols at https://plos.org/protocols?utm_medium=editorial-email&utm_source=authorletters&utm_campaign=protocols.

As the corresponding author, your ORCID iD is verified in the submission system and will appear in the published article. PLOS supports the use of ORCID, and we encourage all coauthors to register for an ORCID iD and use it as well. Please encourage your coauthors to verify their ORCID iD within the submission system before final acceptance, as unverified ORCID iDs will not appear in the published article. *Only*  the individual author can complete the verification step; PLOS staff *cannot*  verify ORCID iDs on behalf of authors.

We look forward to receiving your revised manuscript.

Kind regards,

Latha Marasamy, Ph.D

Academic Editor

PLOS One

Journal Requirements:

When submitting your revision, we need you to address these additional requirements.

1.Please ensure that your manuscript meets PLOS ONE's style requirements, including those for file naming. The PLOS ONE style templates can be found at

https://journals.plos.org/plosone/s/file?id=wjVg/PLOSOne_formatting_sample_main_body.pdf and

https://journals.plos.org/plosone/s/file?id=ba62/PLOSOne_formatting_sample_title_authors_affiliations.pdf

2. Please note that PLOS One has specific guidelines on code sharing for submissions in which author-generated code underpins the findings in the manuscript. In these cases, we expect all author-generated code to be made available without restrictions upon publication of the work. Please review our guidelines at https://journals.plos.org/plosone/s/materials-and-software-sharing#loc-sharing-code and ensure that your code is shared in a way that follows best practice and facilitates reproducibility and reuse.

3. In the online submission form you indicate that your data is not available for proprietary reasons and have provided a contact point for accessing this data. Please note that your current contact point is a co-author on this manuscript. According to our Data Policy, the contact point must not be an author on the manuscript and must be an institutional contact, ideally not an individual. Please revise your data statement to a non-author institutional point of contact, such as a data access or ethics committee, and send this to us via return email. Please also include contact information for the third party organization, and please include the full citation of where the data can be found.

4. Please ensure that you refer to Figure 1 and 2 in your text as, if accepted, production will need this reference to link the reader to the figure.

5. If the reviewer comments include a recommendation to cite specific previously published works, please review and evaluate these publications to determine whether they are relevant and should be cited. There is no requirement to cite these works unless the editor has indicated otherwise.

[Note: HTML markup is below. Please do not edit.]

Reviewer's Responses to Questions

**Comments to the Author**

1. Is the manuscript technically sound, and do the data support the conclusions?

Reviewer #1: Yes

Reviewer #2: Partly

**********

2. Has the statistical analysis been performed appropriately and rigorously?

Reviewer #1: Yes

Reviewer #2: I Don't Know

**********

3. Have the authors made all data underlying the findings in their manuscript fully available?

The PLOS Data policy

Reviewer #1: Yes

Reviewer #2: Yes

**********

4. Is the manuscript presented in an intelligible fashion and written in standard English?

Reviewer #1: Yes

Reviewer #2: Yes

**********

Reviewer #1: Reviewer Comments

Lack of Experimental Validation

The study is entirely based on analytical simulations performed in MATLAB without any experimental verification. While the theoretical comparison is valuable, the absence of experimental data significantly limits the practical relevance of the findings. The authors are encouraged to validate their results with published experimental data or provide a critical comparison with literature-reported values.

Insufficient Justification of Model Parameters

Key parameters such as interface state density (Nss), standard deviation (σ), and inhomogeneity parameter (γ) are central to the analysis. However, the manuscript does not clearly justify the selected parameter ranges or their physical relevance to real SiC devices. A more detailed explanation or literature support is necessary.

Limited Novelty in Model Comparison

The three models (Cowley and Sze, Werner, and Tung) are well-established in the literature. The manuscript should more clearly highlight the novelty of this comparative study, particularly how it advances beyond existing reviews or prior comparative works.

Inadequate Physical Interpretation

Although the models are described, the discussion lacks depth in explaining the underlying physics of the observed trends. The manuscript would benefit from a more rigorous interpretation of how each parameter influences carrier transport and barrier formation in different SiC polytypes.

Absence of Temperature-Dependent Analysis

Schottky barrier inhomogeneity is strongly temperature dependent, especially in SiC-based power devices. The manuscript does not consider temperature effects, which is a significant limitation. Including temperature-dependent simulations would greatly strengthen the study.

Comparison Between SiC Polytypes Needs Strengthening

The conclusion that all three SiC polytypes show similar trends is not sufficiently supported with detailed quantitative comparison. The authors should provide clearer comparative plots, tabulated results, or normalized analysis to substantiate this claim.

Practical Device Implications Are Underdeveloped

While the manuscript mentions applications in high-temperature power devices, the discussion does not adequately connect the modeling results to real device design considerations (e.g., contact engineering, reliability, leakage current control). This aspect should be expanded.

Clarity in MATLAB Simulation Methodology

The manuscript lacks sufficient detail regarding the simulation procedure, boundary conditions, and assumptions used in MATLAB. Reproducibility requires a clearer description of equations, numerical methods, and convergence criteria.

Graphical and Data Presentation

The effectiveness of the comparative analysis depends heavily on visual representation. The manuscript should include clearer, high-quality plots showing I–V characteristics, SBH variation, and parameter sensitivity across models and polytypes.

Language and Technical Presentation

The manuscript contains several long and complex sentences that affect readability. Careful language editing is required to improve clarity, precision, and flow of the discussion.

Reviewer #2: The novelty of the work must be included in the introduction part.

The current–voltage (I–V) characteristics were simulated at 100 K, 300 K, and 500 K for each SiC polytype (3C, 4H, and 6H). Why did the authors choose these temperatures? Please justify.

Although a conclusion is provided for the comparison between the 3C, 4H, and 6H models, the purpose of the comparison study is not clear. Please include it.

How reliable is the MATLAB simulation software, and how will this study be useful for experimental investigations?

Please check the alignments.

**********

what does this mean?). If published, this will include your full peer review and any attached files.

If you choose “no”, your identity will remain anonymous but your review may still be made public.

**Do you want your identity to be public for this peer review?**  For information about this choice, including consent withdrawal, please see our Privacy Policy

Reviewer #1: **Yes:**  Dr.P.Sasikumar

Reviewer #2: No

**********

[NOTE: If reviewer comments were submitted as an attachment file, they will be attached to this email and accessible via the submission site. Please log into your account, locate the manuscript record, and check for the action link "View Attachments". If this link does not appear, there are no attachment files.]

To ensure your figures meet our technical requirements, please review our figure guidelines: https://journals.plos.org/plosone/s/figures

You may also use PLOS’s free figure tool, NAAS, to help you prepare publication quality figures: https://journals.plos.org/plosone/s/figures#loc-tools-for-figure-preparation.

NAAS will assess whether your figures meet our technical requirements by comparing each figure against our figure specifications.

Attachment Submitted filename: Reviewer comments-D-26-11447.docx

# Author comment: Author response to Decision Letter 1

**DOI:** 10.1371/journal.pone.0355480.r002

2 Jun 2026

Manuscript Number: PONE-D-26-11447

Response to Reviewers

We would like to thank the Editor and the Reviewers for their valuable time and constructive comments regarding our manuscript entitled “Comparison of Inhomogeneous Schottky Barrier Modeling Approaches in SiC Polytypes” submitted to PLOS ONE. We sincerely appreciate the insightful suggestions and comments provided by the Reviewers, which helped us improve the quality and clarity of the manuscript. We have carefully addressed all comments and incorporated the corresponding revisions in the updated version of the paper.All modifications made in the revised manuscript are highlighted in red. A detailed point-by-point response to each reviewer comment is provided below in blue.

1.Lack of Experimental Validation

The study is entirely based on analytical simulations performed in MATLAB without any experimental verification. While the theoretical comparison is valuable, the absence of experimental data significantly limits the practical relevance of the findings. The authors are encouraged to validate their results with published experimental data or provide a critical comparison with literature-reported values.

Author reponse :

We sincerely thank the reviewer for this valuable suggestion. We agree that comparing theoretical modeling with experimental results is important to demonstrate the physical relevance of our study. In the revised manuscript, we improved the discussion by directly comparing our simulation results with previously reported experimental studies as folluws :

Cowley–Sze Validation (Nss): The simulated decrease in Nss with increasing temperature has been cross-referenced with the experimental work of Benamara et al. [29] on SiC Schottky diodes and the TCAD simulations of Toumi et al. [30] on GaAs structures, both confirming the thermal emission of trapped charges.

Werner Model Validation (σ Trends): Our simulated temperature dependence of the effective barrier height is in rigorous agreement with the experimental I-V-T characteristics reported by Ouennoughi et al [38] on Mo/4H-SiC Schottky diodes.

Tung Model Validation (γ): The simulated carrier-channeling at low temperatures (Figure 8) and the barrier lowering via the "pinch-off" effect (Figure 9) accurately replicate the experimental extractions of Omar et al. [20] on Ni-Si/4H-SiC diodes, confirming the validity of our local potential fluctuation modeling.

Current-Voltage (I-V) Validation: To secure a final layer of quantitative validation, the room-temperature forward I-V characteristics simulated in Figure 10 show excellent visual and quantitative slope alignment with the experimental data from high-voltage (4.5 kV) Ni/4H-SiC diodes published by Bluet et al[24].

By aligning our MATLAB frameworks with these diverse experimental and numerical studies, we ensure that our comparative analysis accurately reflects the physical transport dynamics of real-world SiC devices.

Changes in the Manuscript:

Sections 3.1.1, 3.1.2, 3.1.3 (Pages 11,15 and 20 ): New comparative discussions have been integrated to explicitly link Figures 3, 6 and 8 with the experimental data of Benamara et al.[29] Ouennoughi et al[38] and Omar et al.[20]

Section 3.3 (Page 23): A dedicated text insertion has been made to highlight the quantitative agreement between Figure 10 and the experimental data of Bluet et al[24].

2-Insufficient Justification of Model Parameters

Key parameters such as interface state density (Nss), standard deviation (σ), and inhomogeneity parameter (γ) are central to the analysis. However, the manuscript does not clearly justify the selected parameter ranges or their physical relevance to real SiC devices. A more detailed explanation or literature support is necessary.

Author reponse :

We thank the reviewer for highlighting the need for a clearer justification of our parameters.

2.1 Justification of the standard deviation value (σ)

In the revised manuscript, we have set the standard deviation to σ = 0.09eV. This choice is not arbitrary but is based on a rigorous survey of experimental extractions from the literature for SiC Schottky diodes:

Ouennoughi et al.[38] reported a value of 0.08 eV for Mo/4H-SiC contacts.

Bluet et al.[24] obtained 0.09 eV for high-voltage Ni/4H-SiC diodes.

Toumi et al.[37] extracted 0.092 eV for similar SiC-based structures.

By selecting 0.09 eV as a representative mean value, our model gains physical credibility and aligns perfectly with the current state of experimental research. We believe this firmly addresses the concern regarding the physical relevance of our simulation parameters.

2.2. Justification of the Inhomogeneity Parameter Range (γ):

The selected range for the patch inhomogeneity parameter, 2×10-4 to 8× 10-4 cm^2/3^V^1/3^, is directly based on the values reported in foundational and experimental literature:

Tung et al. [19]: analyzed the Schottky barrier inhomogeneities across a γ range of 2×10-4 to 4× 10-4 cm^2/3^V^1/3^.

Omar et al. [20]: extracted the Schottky barrier parameters for γ values spanning from 2×10-3 to 7× 10-4 cm^2/3^V^1/3^ on Ni-Si/4H-SiC interfaces.

Our simulation range 2×10-4 to 8× 10-4 cm^2/3^V^1/3^ was established directly on this basis to cover these theoretical and experimental boundaries while accounting for slightly higher potential fluctuations.

2.3 Physical Justification of the Nss Values

We agree with the reviewer that the range of the interface state density must reflect real device characteristics. To ensure strict physical relevance to the studied material, the selected range for our Nss analysis (5×1011 to 5×1013 cm-2 eV-1) has been validated against the experimental work of Khanna et al.[32], who specifically investigated metal/4H-SiC Schottky interfaces and Benamara et al.[29].

Khanna et al. reported extracted Nss values in the range of 2×1012 to 6.5×1012 cm-2 eV-1. And Benamara et al., who worked on the Ni/6H-SiC diode, reported a reduction in the interface state density Nss from 1.2×1013 to 6×1012 cm−2eV−1. Our selected range directly encompasses these experimental values.

Changes in the Manuscript:

Section 3.1.2 (page 15): A new paragraph has been added to justify the choice of σ = 0.09 eV by referencing the experimental works of Ouennoughi et al.[37], Bluet et al.[24], and Toumi et al [36].

Section 3.2 (page 19) : The text has been updated to explicitly specify that our simulation range is built upon the respective ranges of Tung et al.[19] and Omar et al.[20].

Section 3.1.1 (page 13) : The text has been updated to explicitly justify the selected Nss range by referencing the experimental boundaries established by Khanna et al.[32] for 4H-SiC structures and Benamara et al. [29].

3-Limited Novelty in Model Comparison

The three models (Cowley and Sze, Werner, and Tung) are well-established in the literature. The manuscript should more clearly highlight the novelty of this comparative study, particularly how it advances beyond existing reviews or prior comparative works.

Author reponse:

We completely understand the reviewer's concern regarding the well-established nature of the individual models. However, we would like to clarify that the novelty of this work does not lie in the development of new analytical formulations, but rather in the simultaneous quantitative benchmark and unified mapping of these frameworks applied to SiC polytypes (4H, 6H, and 3C). While existing literature typically reviews these models qualitatively or applies a single model to fit specific experimental data, our study advances prior works in three distinct ways:

Unified Multi-Model Confrontation : We implement a side-by-side quantitative comparison of the interface-state model (Cowley-Sze), the macroscopic fluctuation model (Werner), and the microscopic patch model (Tung) under identical simulation boundaries. This directly identifies the exact temperature and voltage thresholds where each model loses its physical validity.

Polytype-Specific Sensitivity Mapping : We systematically correlate the variation of inhomogeneity parameters (σ, γ and Nss) with the physical properties of different SiC polytypes, providing a clear reference grid for device designers to know which model is most accurate depending on the specific polytype and defect density.

Transition Dynamics : Our framework clearly demonstrates how microscopic potential patches (Tung) macroscopically average out into a Gaussian distribution (Werner) as a function of temperature, bridging the gap between these two theories in a way that standard reviews do not quantify.

We have significantly revised the Introduction and Conclusion sections to explicitly highlight these contributions and clearly state how this study provides a predictive tool rather than a simple literature review

Changes in the Manuscript:

Section 1 (Introduction, page 3) : The paragraph preceding Figure 1. has been rewritten to explicitly frame the novelty, objectives, and distinct advantages of this comparative framework over existing literature.

Section 4 (Conclusion, page 33) : We have revised the conclusion to emphasize the practical engineering insights gained from this simultaneous multi-model evaluation.

4-Inadequate Physical Interpretation

Although the models are described, the discussion lacks depth in explaining the underlying physics of the observed trends. The manuscript would benefit from a more rigorous interpretation of how each parameter influences carrier transport and barrier formation in different SiC polytypes.

Author reponse :

We thank the reviewer for this insightful comment. We agree that strengthening the physical interpretation of the carrier transport mechanisms across the different SiC polytypes enhances the depth of the paper. In the revised manuscript, we have expanded the discussion sections to explicitly connect our simulation parameters (σ, γ and Nss) with the intrinsic semiconductor physics of 4H, 6H, and 3C-SiC:

Standard deviation (σ): We have clarified that the temperature dependence of the effective barrier height (Φ_B) is governed by the competitive trade-off between thermal energy and the standard deviation (σ). At low temperatures, carriers lack sufficient energy to surmount the nominal barrier and are forced to flow through localized patches with lower potential energy. This effect is more pronounced in wider bandgap polytypes (4H-SiC, Eg = 3.26 eV) compared to lower bandgap polytypes (3C-SiC, Eg = 2.3 eV), as the relative magnitude of potential fluctuations impacts carrier distribution more severely when the ideal barrier height is larger [34][35] [36].

inhomogeneity parameter (γ): We have added a comprehensive explanation of the pinch-off effect dictated by the inhomogeneity parameter γ. When γ increases, thelateral electric field arising from the surrounding high-barrier regions strongly interacts with the low-barrier patches. This electrostatic interaction modulates the potential profile, altering the effective transmission coefficient of the carriers and deviating the transport from the pure thermionic emission (TE) theory [8][20][41].

Interface states (Nss): We have enriched the text to discuss how the variations in Nss across the polytypes directly influence the degree of Fermi-level pinning. Higher calculated Nss values signify a greater screen of the metal work function by the interface traps, which severely limits the control of the barrier formation by the choice of the contact metal [6].

Changes in the Manuscript:

Sections 3.1.1, 3.1.2, and 3.1.3 (Pages 11,14 ,18,19) : Detailed paragraphs containing these physical interpretations and transport dynamics have been integrated to accompany each respective simulation curve.

5-Absence of Temperature-Dependent Analysis

Schottky barrier inhomogeneity is strongly temperature dependent, especially in SiC-based power devices. The manuscript does not consider temperature effects, which is a significant limitation. Including temperature-dependent simulations would greatly strengthen the study.

Author reponse :

We thank the reviewer for this valuable comment. To make the temperature-dependent analysis more explicit and easier to identify, a new section entitled “Comparative Temperature-Dependent Analysis” has been added in the revised manuscript. This section provides a dedicated discussion and a summary table highlighting the influence of temperature on the effective Schottky barrier height in the Cowley–Sze, Werner, and Tung models.In addition, we would like to clarify that temperature-dependent simulations were already included in the original manuscript. Specifically, Figures 3, 4, 6, and 8 present the variation of the interface state density and effective Schottky barrier height as a function of temperature, while Figures 13–15 show the corresponding I–V characteristics at 100 K, 300 K, and 500 K for all three SiC polytypes.

Model Temperature dependence of effective SBH Physical origin

Cowley and Sze Slight increase for Pt/3C-SiC and Ni/4H-SiC; decrease for Ti/6H-SiC Variation of interface-state pinning and bandgap

Werner Monotonic increase Reduced dominance of low-barrier regions

Tung Decrease from 100 to 300 K, then saturation Activation of localized low-barrier patches

Changes in the Manuscript:

An additional section (Section 3.1.4, page 21) was dedicated to the role of temperature and its effect on the three inhomogeneous models, including the addition of a comparative table (Table 2).

6-Comparison Between SiC Polytypes Needs Strengthening

The conclusion that all three SiC polytypes show similar trends is not sufficiently supported with detailed quantitative comparison. The authors should provide clearer comparative plots, tabulated results, or normalized analysis to substantiate this claim.

Author reponse

We thank the reviewer for this valuable comment. To provide a clearer and more quantitative comparison, we have added Table 3, entitled “Quantitative Comparison of Forward Current Density at 1.5 V for the Three Inhomogeneity Models and SiC Polytypes at Different Temperatures.” This table summarizes the forward current density values at 1.5 V for all three SiC polytypes (3C-SiC, 4H-SiC, and 6H-SiC) at 100 K, 300 K, and 500 K. The added quantitative results show that, although significant differences exist in the absolute current-density values, all three SiC polytypes exhibit a consistent thermally activated behavior within each inhomogeneity model. In particular, the Werner and Tung models predict a progressive increase in forward current with temperature for all investigated polytypes. These results confirm that the predicted electrical behavior is influenced more strongly by the underlying inhomogeneity model than by the SiC polytype itself

Model Polytype Forward Current density at 1.5 V and 100K (A /cm2) Forward Current density at 1.5 V and 300K (A /cm2) Forward Current density at 1.5 V and 500K (A /cm2)

Cowley–Sze 3C-SiC 1,8×10-10 7×10-6 4×10-2

Cowley–Sze 4H-SiC 1,6×10-10 6×10-10 4×10-2

Cowley–Sze 6H-SiC 2×10-2 11×10-2 24×10-2

Werner 3C-SiC 1×10-3 4×10-2 0,16

Werner 4H-SiC 1,53×10-10 3×10-3 4×10-2

Werner 6H-SiC 19×10-2 2×10-1 54×10-1

Tung 3C-SiC 1×10-3 4×10-2 0,18

Tung 4H-SiC 1,6×10-10 7×10-3 4×10-2

Tung 6H-SiC 26×10-2 32×10-2 5×10-1

Changes in the Manuscript:

Sections 3.3 (Page 32) : We have added Table 3, which presents a comparison of the forward current density values for the three inhomogeneity models.

7-Practical Device Implications Are Underdeveloped

While the manuscript mentions applications in high-temperature power devices, the discussion does not adequately connect the modeling results to real device design considerations (e.g., contact engineering, reliability, leakage current control). This aspect should be expanded.

Author reponse :

Thank you for this valuable comment. In response, we have added a new subsection entitled “Practical Device Implications” in the Discussion section, immediately before the Conclusion. This subsection expands the connection between the modeling results and practical design considerations for high-temperature SiC power devices.

The added discussion explains how the choice of transport model affects the predicted Schottky barrier height and current transport characteristics, and how these differences directly influence key device par

Attachment Submitted filename: reponse to reviewers.docx

# Peer review: Decision Letter 1

**DOI:** 10.1371/journal.pone.0355480.r003

**Authors:** Latha Marasamy

22 Jul 2026

Comparison of Inhomogeneous Schottky Barrier Modeling Approaches in SiC Polytypes

PONE-D-26-11447R1

Dear Dr. Mustapha Habib,

We’re pleased to inform you that your manuscript has been judged scientifically suitable for publication and will be formally accepted for publication once it meets all outstanding technical requirements.

Within one week, you’ll receive an e-mail detailing the required amendments. When these have been addressed, you’ll receive a formal acceptance letter and your manuscript will be scheduled for publication.

An invoice will be generated when your article is formally accepted. Please note, if your institution has a publishing partnership with PLOS and your article meets the relevant criteria, all or part of your publication costs will be covered. Please make sure your user information is up-to-date by logging into Editorial Manager at Editorial Manager® and clicking the ‘Update My Information' link at the top of the page. For questions related to billing, please contact billing support.

If your institution or institutions have a press office, please notify them about your upcoming paper to help maximize its impact. If they’ll be preparing press materials, please inform our press team as soon as possible -- no later than 48 hours after receiving the formal acceptance. Your manuscript will remain under strict press embargo until 2 pm Eastern Time on the date of publication. For more information, please contact onepress@plos.org.

Kind regards,

Latha Marasamy, Ph.D

Academic Editor

PLOS One

Additional Editor Comments (optional):

Reviewers' comments:

Reviewer's Responses to Questions

**Comments to the Author**

Reviewer #2: All comments have been addressed

Reviewer #3: All comments have been addressed

**********

2. Is the manuscript technically sound, and do the data support the conclusions?

Reviewer #2: Yes

Reviewer #3: Partly

**********

3. Has the statistical analysis been performed appropriately and rigorously?

Reviewer #2: I Don't Know

Reviewer #3: Yes

**********

4. Have the authors made all data underlying the findings in their manuscript fully available?

The PLOS Data policy

Reviewer #2: Yes

Reviewer #3: Yes

**********

5. Is the manuscript presented in an intelligible fashion and written in standard English?

Reviewer #2: Yes

Reviewer #3: Yes

**********

Reviewer #2: (No Response)

Reviewer #3: (No Response)

**********

what does this mean?). If published, this will include your full peer review and any attached files.

If you choose “no”, your identity will remain anonymous but your review may still be made public.

**Do you want your identity to be public for this peer review?**  For information about this choice, including consent withdrawal, please see our Privacy Policy

Reviewer #2: No

Reviewer #3: No

**********

# Editor report: Acceptance letter

**DOI:** 10.1371/journal.pone.0355480.r004

**Authors:** Latha Marasamy

PONE-D-26-11447R1

PLOS One

Dear Dr. Habib,

I'm pleased to inform you that your manuscript has been deemed suitable for publication in PLOS One. Congratulations! Your manuscript is now being handed over to our production team.

At this stage, our production department will prepare your paper for publication. This includes ensuring the following:

* All references, tables, and figures are properly cited

* All relevant supporting information is included in the manuscript submission,

* There are no issues that prevent the paper from being properly typeset

You will receive further instructions from the production team, including instructions on how to review your proof when it is ready. Please keep in mind that we are working through a large volume of accepted articles, so please give us a few days to review your paper and let you know the next and final steps.

Lastly, if your institution or institutions have a press office, please let them know about your upcoming paper now to help maximize its impact. If they'll be preparing press materials, please inform our press team within the next 48 hours. Your manuscript will remain under strict press embargo until 2 pm Eastern Time on the date of publication. For more information, please contact onepress@plos.org.

You will receive an invoice from PLOS for your publication fee after your manuscript has reached the completed accept phase. If you receive an email requesting payment before acceptance or for any other service, this may be a phishing scheme. Learn how to identify phishing emails and protect your accounts at https://explore.plos.org/phishing.

If we can help with anything else, please email us at customercare@plos.org.

Thank you for submitting your work to PLOS ONE and supporting open access.

Kind regards,

PLOS ONE Editorial Office Staff

on behalf of

Dr. Latha Marasamy

Academic Editor

PLOS One
